# Efficacy and safety of intralymphatic immunotherapy for allergic rhinitis: an overview of systematic reviews and meta analyses

**DOI:** 10.3389/fmed.2025.1709531

**Published:** 2025-12-05

**Authors:** Zhuang Wang, Xiaofei Xie, Zhikai Qiu, Dongze Li, Yongfu Song, Na Wang, Bing Tian, Yongji Wang

**Affiliations:** 1 College of Traditional Chinese Medicine, Changchun University of Chinese Medicine, Changchun, Jilin, China; 2 Department of Pediatric Internal Medicine One, Affiliated Hospital to Changchun University of Chinese Medicine, Changchun, Jilin, China; 3 Department of Pediatrics, Jilin Academy of Chinese Medicine Sciences, Changchun, Jilin, China

**Keywords:** allergic rhinitis, efficacy, intralymphatic immunotherapy, overview of systematic reviews and meta analyses, safety

## Abstract

**Objective:**

Conduct an overview of systematic reviews and meta-analyses on intralymphatic immunotherapy for allergic rhinitis, providing systematic evidence to optimize clinical practice and evidence-based decision-making.

**Methods:**

A computer-based retrieval system was used to comprehensively search databases such as PubMed, Embase, Cochrane Library, Web of Science, CNKI, VIP, WANFANG, and CBM. The retrieval time limit was set from the inception date of each database to August 14, 2025, aiming to obtain systematic review/Meta-analysis literatures on lymph node intralymphatic immunotherapy for the treatment of allergic rhinitis. Use evaluation tools such as ROBIS, AMSTAR-2, PRISMA 2020, and GRADE to perform quality re-evaluations on the systematic reviews/Meta-analyses of included studies from the aspects of bias risk, methodology, reporting, and evidence level, and conduct comprehensive re-evaluations on the outcome indicators of the included studies.

**Results:**

A total of seven systematic reviews/meta-analyses were included. Risk of bias assessment indicated all studies had low risk. Methodological quality evaluation revealed six studies were of low quality and one was of very low quality. In terms of reporting quality, all included studies demonstrated high quality, with PRISMA scores ranging from 33 to 40. For outcome measures, evidence quality assessment identified 6 high-quality, 13 moderate-quality, 18 low-quality, and 27 very low-quality results. Quantitative analysis showed that intralymphatic immunotherapy not only improved subjective symptom scores in allergic rhinitis patients but also effectively induced immune tolerance. Qualitative analysis further confirmed that this targeted approach significantly enhanced allergen tolerance and reduced nasal symptom severity. The therapy demonstrated a favorable safety profile, primarily characterized by mild local adverse events, with substantially fewer systemic reactions compared to conventional subcutaneous immunotherapy.

**Conclusion:**

Intralymphatic immunotherapy demonstrates efficacy in improving symptoms and objective indicators of allergic rhinitis, particularly for grass pollen and mixed allergens, with acceptable short-to-medium term outcomes despite limited long-term effectiveness. The treatment exhibits a favorable safety profile dominated by mild local reactions. However, these findings are constrained by low evidence quality. Future studies should adhere to established guidelines to produce higher-quality evidence through systematic reviews and meta-analyses.

**Systematic review registration:**

The systematic review was registered on PROSPERO (https://www.crd.york.ac.uk/PROSPERO/) with the registration number CRD420251126501.

## 1 Introduction

Allergic rhinitis (AR), a chronic IgE-mediated inflammatory disease of the nasal mucosa (1), affects 10%–30% of the global population (2). This non-infectious condition results from the interplay of genetic predisposition, environmental factors, and allergen exposure (3, 4). The pathogenic process evolves through three distinct phases: sensitization, provocation, and chronic inflammation (1, 5). Clinically, it is characterized by nasal-ocular symptoms and systemic manifestations (6), which can lead to various complications such as asthma and conjunctivitis (7), affecting the patient’s quality of life and health (8). The current treatment system includes allergen avoidance, drug intervention, immunotherapy, and the application of biological agents (9). Nasal corticosteroids, antihistamines, and leukotriene receptor antagonists are the first-line treatment options, while immunotherapy is a key means to alter the disease course (1). As the only therapy currently capable of altering the natural course of allergic diseases (10, 11), the core value of specific immunotherapy (SIT) lies in blocking abnormal immune responses at their root through immune regulation against specific allergens. Traditional subcutaneous immunotherapy (SCIT) and sublingual immunotherapy (SLIT) have obtained clear clinical evidence, but are limited by bottlenecks such as long treatment cycles and inconvenient treatment processes (12). Therefore, there is a need to explore new methods for SIT of AR.

Intralymphatic immunotherapy (ILIT) represents a significant advancement in the field of allergy treatment. By precisely injecting low-dose allergens into the lymphatic vessels, it can directly act on the key nodes of the immune system, enabling more efficient “retraining” of the immune system and thus fundamentally reducing allergic reactions (13–15). Compared to traditional SCIT and SLIT, ILIT offers advantages of shorter treatment cycles and lower dosage requirements (16). In the research and application fields, a Swiss research team first conducted human ILIT clinical trials in 2000 (17). After more than 20 years of exploration, clinical trial data now support its safety, tolerability, and efficacy (18–23). However, it remains difficult to comprehensively evaluate its real-world effects and potential risks across different regions, populations, and long-term use (24). In recent years, although several systematic reviews/meta-analyses (SAs/MAs) have investigated the efficacy of ILIT for AR, these studies exhibit significant heterogeneity in methodological design, evaluation dimensions, and conclusions. This study is the first to adopt the highest-level evidence synthesis method—an overview of systematic reviews and meta-analyses—to comprehensively collate and evaluate all SAs/MAs in the field of ILIT for AR. Through this research, we aim to construct a panoramic “evidence map” of ILIT for AR, addressing the gaps in indirect comparisons found in previous studies. By conducting a rigorous quality assessment, we seek to clarify the strengths and limitations of existing evidence, clearly delineate which questions are sufficiently supported by evidence and which areas still lack high-quality SAs/MAs. This work aims to provide higher-level evidence-based medical support for clinical practice and offer important references for future research directions.

## 2 Materials and methods

### 2.1 Protocol registration

The protocol registration of this study was completed in PROSPERO before the commencement of the research, and the registration number is CRD420251126501.

### 2.2 Inclusion criteria

(1) The various SAs/MAs with ILIT as the main intervention for the treatment of AR or allergic rhinoconjunctivitis (AR and allergic rhinoconjunctivitis are highly interconnected in terms of etiology, pathogenesis, diagnosis, and treatment (25–28). The signs and symptoms of allergic rhinoconjunctivitis encompass those of AR. To ensure the comprehensiveness of the study, SAs/MAs on allergic rhinoconjunctivitis were also included in the research on AR). (2) ILIT as the main intervention in the treatment group and placebo, conventional treatment and other SIT as the main intervention in the control group. (3) The included SAs/MAs were restricted to randomized controlled trials (RCTs) as primary studies. (4) The participants and/or doctors, and/or both are unaware of the allocation of immunotherapy or placebo (single-blind, double-blind, or unblinded). (5) The participants are randomly or non-randomly assigned to the treatment group or the control group.

### 2.3 Exclusion criteria

(1) Repeated literature. (2) Literature for which the full text or complete data cannot be obtained. (3) Synthesis or meeting summaries. (4) Deviation from the research topic.

### 2.4 Outcome indicator

(1) Symptom score. (2) Medication score. (3) Combined symptom and medication score (CSMs). (4) Visual analog scale (VAS). (5) Adverse events or adverse reactions. (6) Quality of life.

### 2.5 Search strategy

Search all SAs/MAs literature on the treatment of AR or allergic rhinoconjunctivitis by ILIT from the establishment of the databases to August 14, 2025 in eight databases, namely PubMed, Embase, Cochrane Library, Web of Science, CNKI, VIP, WANFANG and CBM, through the computer network. The search terms and search strategy are as follows (taking Embase as an example):

#1:‘allergic rhinitis’/exp.
#2:‘rhinitis, allergic’.
#3:‘allergic rhinitis’.
#4:‘hypersensitive rhinitis’.
#5:‘anaphylactic rhinitis’.
#6:‘nasal allergy’.
#7:rhinallergosis.
#8:#1 OR #2 OR #3 OR #4 OR #5 OR #6 OR #7.
#9:‘Intralymphatic immunotherapy’.
#10:‘Intralymphatic immunization’.
#11:#9 OR #10.
#12:‘meta analysis’/exp.
#13:‘systematic review’/exp.
#14:‘meta’.
#15:‘meta analysis’.
#16:‘systematic review’.
#17:#12 OR #13 OR #14 OR #16.
#18:#8 AND #11 AND #17.

### 2.6 Literature screening and data extraction

This study adopted a double-blind, parallel, independent screening and cross-checking mechanism. Two researchers carried out the literature screening and data extraction work in strict accordance with the standardized process. In case of any disagreement, a third researcher with a senior professional title would be invited to intervene and make a decision to ensure the reliability and consistency of the research results. In the initial screening stage, the two researchers independently and systematically evaluated the titles and abstracts of the literature using the double-blind method. Based on the pre-established inclusion and exclusion criteria, the obviously irrelevant studies were excluded. In the re-screening stage, the full-text of the literature retained in the initial screening was deeply reviewed. Through the cross-checking and consensus-discussion mechanism, the studies that met the inclusion criteria were ultimately determined. After completing the literature screening, standardized data extraction was immediately conducted. The extracted data included the first author, publication year, the number of original studies and sample size included in the SAs/MAs, intervention and control measures, quality evaluation tools, outcome indicators, main conclusions, and other information.

### 2.7 Quality assessment

#### 2.7.1 Risk of bias assessment

We assessed the risk of bias in the included SAs/MAs using the Risk of Bias in Systematic Reviews (ROBIS) (29). The implementation process consists of three progressive stages: first evaluating the relevance of the systematic review to the research question, then determining the degree of bias risk during the systematic review development process, and finally judging the overall risk of bias level. For each stage, evaluators must respond to key signaling questions by selecting from four options: “yes,” “probably yes,” “no,” or “probably no/no information.” Ultimately, the risk of bias is classified into three levels: “low risk,” “high risk,” or “unclear,” thereby evaluating potential bias risks in SAs/MAs.

#### 2.7.2 Methodological quality assessment

We evaluated the methodological quality of the included SAs/MAs using the Assessment of Multiple Systematic Reviews-2 (AMSTAR 2) (30, 31). This 16-item scale includes seven critical items (items 2, 4, 7, 9, 11, 13, and 15), with each item rated as “yes” “partial yes” or “no.” An item was considered adequately reported when the combined percentage of “yes” and “partial yes” responses reached 70% or higher. The quality grading followed a hierarchical system: studies with no deficiencies in critical items and ≤3 unmet non-critical items were rated as high quality; those with no critical item deficiencies but ≥4 unmet non-critical items as moderate quality; studies with critical item deficiencies and ≤3 unmet non-critical items as low quality; and those with critical item deficiencies plus ≥4 unmet non-critical items (or complete absence of critical items) as very low quality, thereby ensuring scientifically rigorous and reliable quality assessment outcomes.

#### 2.7.3 Reporting quality assessment

The reporting quality of included SAs/MAs was assessed using the Preferred Reporting Items for Systematic Reviews and Meta-Analyses 2020 (PRISMA 2020) (32, 33). This guideline establishes a comprehensive evaluation framework consisting of 27 core items (with 42 sub-items) organized across seven dimensions: title, abstract, introduction, methods, results, discussion, and other information. During evaluation, researchers strictly assessed each item based on actual reporting completeness using a three-level rating system (“yes” “partial yes” or “no”). An item was deemed adequately reported when the combined percentage of “yes” and “partial yes” ratings reached ≥50%. Scoring was performed as follows: fully reported items (Y) scored 1 point, partially reported items (PY) scored 0.5 points, and unreported items (N) scored 0 points, yielding a maximum possible score of 42 points. Based on total scores, reporting completeness was categorized into three tiers: scores ≥80% (33–42 points) indicated “relatively complete reporting” (high quality); scores between 60% and 80% (25–32 points) indicated “reporting with certain deficiencies” (moderate quality); and scores <60% (<25 points) indicated “substantial information gaps” (low quality).

#### 2.7.4 Evidence quality assessment

The Grading of Recommendations Assessment, Development and Evaluation (GRADE) system was employed to assess the quality of evidence for the pooled outcome effect measures presented in forest plots within the included SAs/Mas (34, 35). A comprehensive evaluation was conducted across five key domains: risk of bias, inconsistency, indirectness, imprecision, and publication bias. Based on the assessment, the evidence quality was precisely categorized into four levels: high (no downgrading), moderate (downgraded by 1 level), low (downgraded by 2 levels), and very low (downgraded by 3 levels or more). Additionally, the effect estimates (including effect size, 95% confidence intervals, I^2^ statistic (%), and *p*-values) for each pooled outcome measure were calculated to ensure the scientific rigor and robustness of the evidence quality assessment.

### 2.8 Quantitative analysis

Structured extraction and validity verification were performed for the SAs/MAs of included studies. Outcome measures that were homogeneous or standardizable in definition, measurement, and reporting were subjected to quantitative synthesis. For dichotomous outcomes, relative risk (RR) with 95% confidence intervals (CI) was used, while standardized mean difference (SMD) was applied for continuous outcomes to eliminate scale variations. Heterogeneity was assessed using a threshold of *I*^2^ ≤ 50% and a Q-test *p* > 0.1; studies meeting these criteria were analyzed using a fixed-effects model, otherwise, a random-effects model was employed. In cases of significant heterogeneity, sensitivity analyses were conducted by sequentially excluding individual studies, followed by stratified subgroup analyses based on population characteristics, intervention dosage, measurement time points, etc. If necessary, meta-regression was performed to identify sources of heterogeneity and adjust the analytical strategy. The final output provided statistically robust and clinically meaningful evidence to support precise evidence-based decision-making.

### 2.9 Qualitative analysis

For outcome measures in the included SAs/MAs that could not be quantitatively analyzed due to heterogeneity, methodological discrepancies, or sample limitations, a qualitative analysis was conducted by initiating a narrative-critical qualitative synthesis procedure. This approach combined narrative summarization with critical appraisal to thoroughly examine textual descriptions, figure/table footnotes, and supplementary materials of outcome measures in the original studies, extracting key findings. Through iterative reading and comparison, scattered findings were clustered into thematic domains. Concurrently, a contrastive analysis was employed to examine the directional trends of conclusions across studies within the same theme, with cross-validation of evidence to identify consensus and controversies. Ultimately, based on the integrated findings, research gaps and limitations were clarified, and future research directions were proposed. This qualitative evidence complements the limitations of quantitative synthesis, provides novel insights, and informs clinical decision-making and subsequent investigations.

## 3 Results and discussion

### 3.1 Results

#### 3.1.1 Results of literature screening

**Figure 1:**
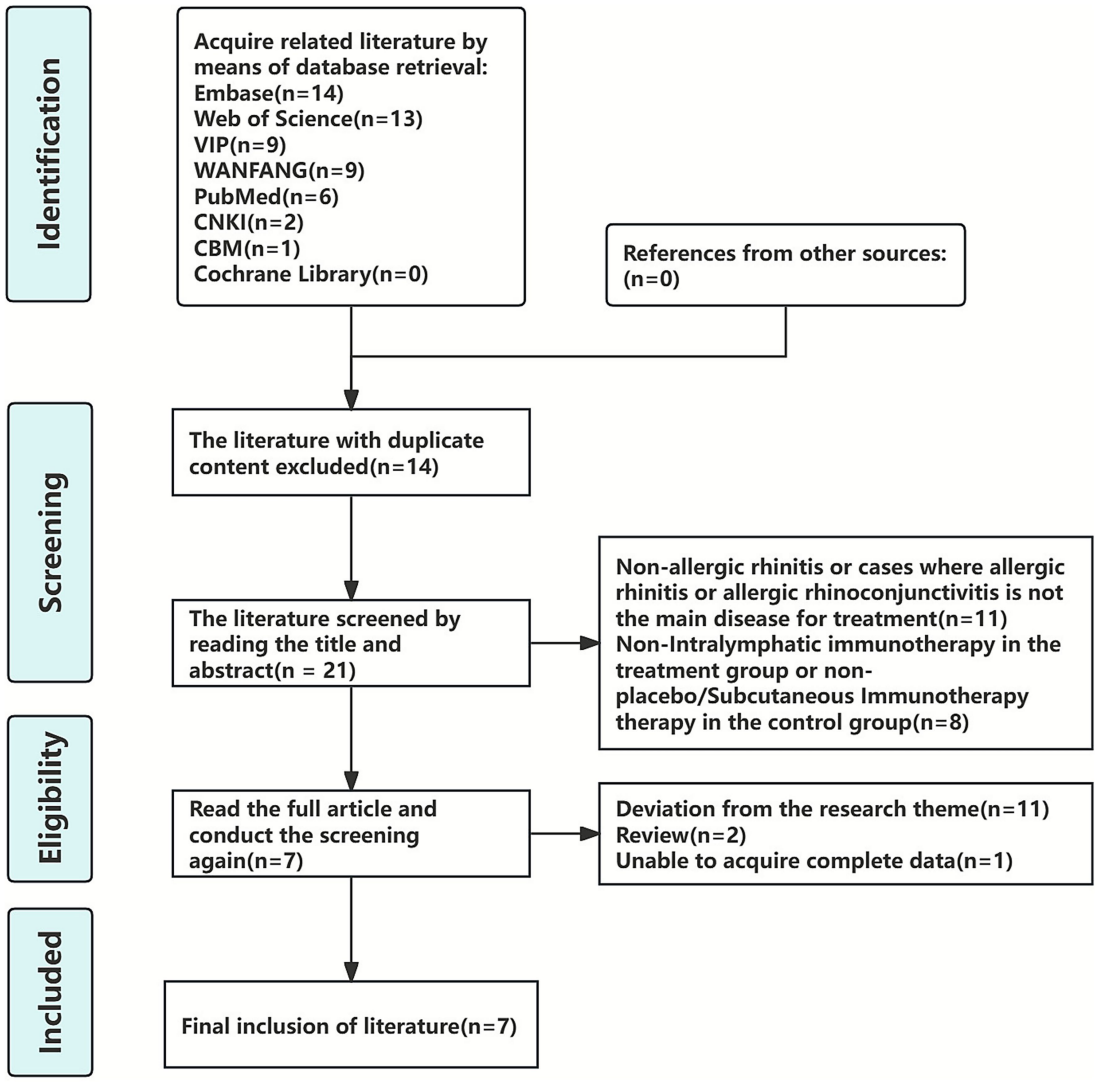
Literature screening process.

Through systematic retrieval, we initially identified 54 potentially relevant articles. After removing duplicates using EndNote X9 software, 40 articles remained for preliminary screening. At this stage, we excluded 11 articles that did not meet the diagnostic criteria for AR, 8 articles with incompatible intervention measures. Subsequently, a full-text review of the remaining 21 articles was conducted, leading to the further exclusion of 11 studies due to irrelevant research topics, 1 article with missing data, and 2 review articles. Ultimately, 7 studies (36–42) that fully met our inclusion criteria were selected for final analysis. The complete screening process is illustrated in Figure 1.

#### 3.1.2 Basic characteristics of the included literature

The analysis included six English-language articles and one Chinese-language article, all published as journal papers between 2021 and 2024. Among the seven included studies, the treatment groups received ILIT while control groups received either placebo or SCIT. Regarding outcome measures: All seven studies reported CSMs (36–42); six studies documented adverse event/reaction (36–40, 42); six studies evaluated quality of life (36–39, 41, 42); five studies separately reported symptom scores and medication scores (36–38, 41, 42); five studies included VAS assessment (37–40, 42); four studies measured serum-specific IgE (36, 38, 41, 42); four studies performed skin prick test (36, 37, 39, 42); three studies conducted subgroup analysis (39, 40, 42); three studies reported nasal provocation test (37, 40, 42); two studies evaluated overall/subjective improvement (36, 40); two studies measured serum-specific IgG4 (38, 42); Other reported outcomes included rescue medication use (36), nasal symptom (41), lymph node symptom (41), compliance (40), and conjunctival provocation test (37). For quality assessment, all seven studies (36–42) utilized the Cochrane Risk of Bias tool. The basic characteristics of included studies are presented in Table 1.

**Table 1:** Characteristics of the included literature.

First author and year	Number of literatures/sample size	Intervention measures		Bias risk measurement tool	Endpoint measure	The main conclusions of the author
		Treatment group	Control group			
Aini et al. (36)	11/452	ILIT	Placebo or SCIT	Cochrane	①②③⑤⑥⑦⑧⑪⑫	ILIT possibly has a role in the treatment of AR patients. This review found it is safe but not effective, which could be contributed by the high variation among the trials.
Werner and Bosso (37)	17/690	ILIT	Placebo	Cochrane	①②③④⑤⑥⑧⑬⑰	ILIT was safe, conferred desensitization to seasonal and nonseasonal allergens, alleviated AR symptoms, and reduced medication use.
Hoang et al. (38)	13/483	ILIT	Placebo or SCIT	Cochrane	①②③④⑤⑥⑦⑩	ILIT showed short-term benefits for seasonal allergic rhinoconjunctivitis. The sustained effects of ILIT were inconclusive. It was well tolerated.
Jiang et al. (39)	13/454	ILIT	Placebo	Cochrane	③④⑤⑥⑧⑨	ILIT might be an alternative immunotherapy strategy for AR patients. Evidence from the current researches validated the safety and effectiveness of ILIT. ILIT was advantageous in improving the clinical symptoms of AR and reducing the need for medications. Moreover, the preseasonal booster injection had a positive impact on CSMs improvement.
Wang et al. (40)	14/582	ILIT	Placebo or SCIT	Cochrane	③④⑤⑨⑫⑬⑯	ILIT was a safe and effective treatment for seasonal allergic rhinoconjunctivitis and could achieve comparable clinical improvement with SCIT with shorter duration and higher compliance. Moreover, ILIT was safer than SCIT.
Liu et al. (41)	11/406	ILIT	Placebo or SCIT	Cochrane	①②③⑥⑦⑭⑮	After the application of the ILIT strategy against AR, the quality of life of patients was improved and the incidence of adverse events associated with nasal symptoms was reduced, but the conclusion needed further verification with more high quality research.
Zeng et al. (42)	9/269	ILIT	Placebo	Cochrane	①②③④⑤⑥⑦⑧⑨⑩⑬	In general, ILIT is safe, but serious complications may occur when the injection dosage is increased, so caution should be exercised. ILIT has significantly improved the VAS for AR induced by grass pollen. Larger-scale and longer-term trials are still needed to standardize the treatment regimen.

①Symptom score; ②Medication score; ③Combined symptom and medication score; ④Visual analog scale; ⑤Adverse even or adverse reaction; ⑥Quality of life; ⑦Serum-specific IgE; ⑧Skin prick test; ⑨Subgroup analysis; ⑩Serum-specific IgG4; ⑪The utilization of rescue medications; ⑫Overall/subjective improvement; ⑬Nasal provocation test; ⑭Nasal symptom; ⑮Lymph node symptom; ⑯Compliance; ⑰Conjunctival provocation test.

#### 3.1.3 Results of the risk of bias assessment

All included studies (36–42) were rated as “passed” in the first phase (applicability assessment) of the ROBIS tool. In the second phase’s four domains (study eligibility criteria, identification and selection of studies, data collection and study appraisal, synthesis and findings) and the third phase (overall risk of bias), all studies were consistently rated as having low risk of bias, with no instances of high risk or unclear risk being identified.

#### 3.1.4 Results of the methodological quality assessment

**Figure 2:**
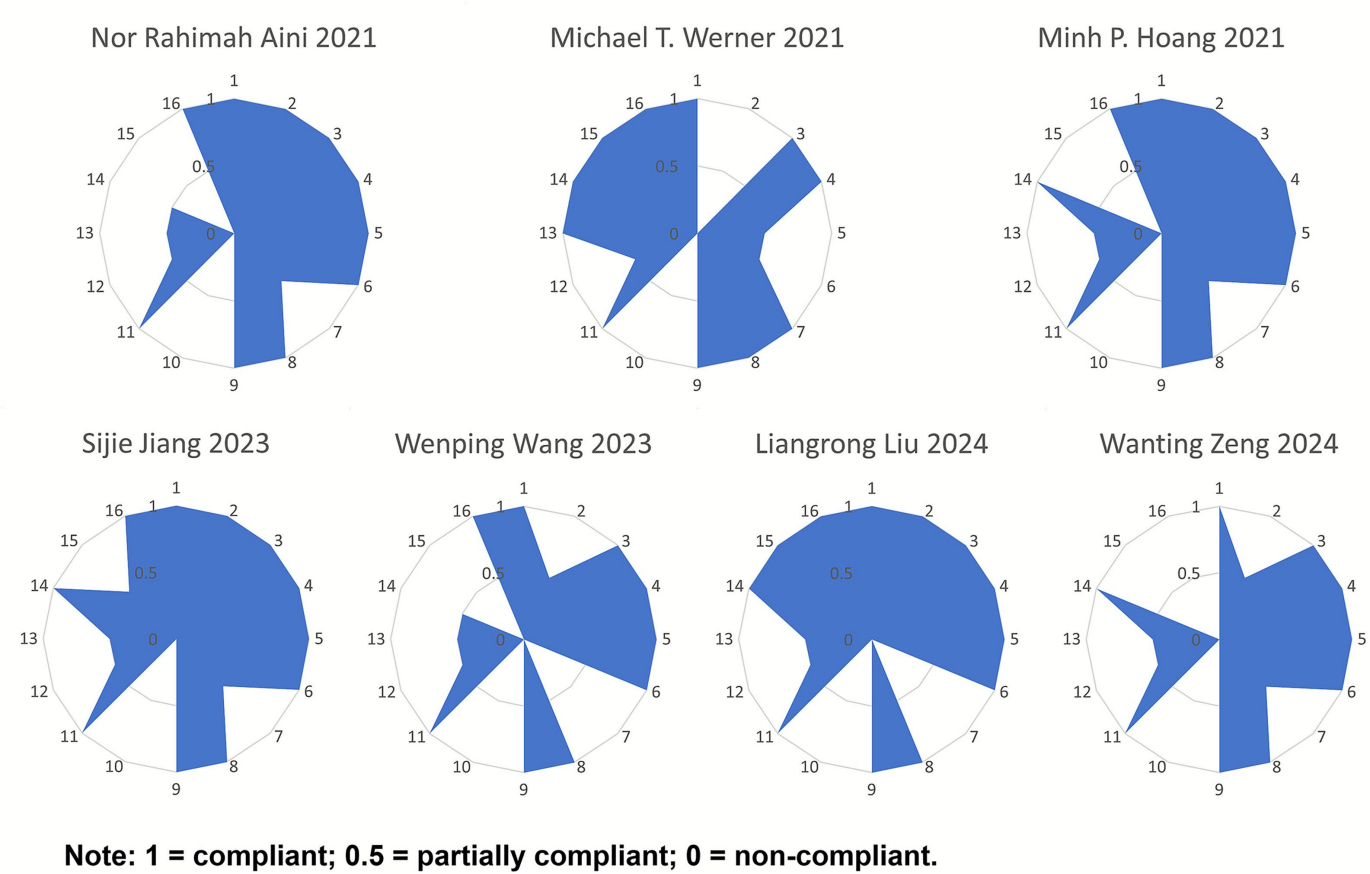
Radar chart of scores for each item of AMSTAR-2.

Among the included studies, six (36, 38–42) were rated as low quality, and one (37) was rated as very low quality. For the key items, all seven studies reported items 4, 9, and 11. Among the remaining key items, the compliance rates were as follows: item 2 (4/7, 57.14%), item 7 (1/7, 14.29%), item 13 (1/7, 14.29%), and item 15 (2/7, 28.57%). For the non-key items, all seven studies reported items 1, 3, and 8, while none reported items 10 and 12. The compliance rates for the remaining non-key items were as follows: item 5 (6/7, 85.71%), item 6 (6/7, 85.71%), item 14 (5/7, 71.43%), and item 16 (6/7, 85.71%). Overall, the methodological quality of the included literature was relatively low. For detailed assessments, refer to Figure 2.

#### 3.1.5 Results of the reporting quality assessment

**Figure 3:**
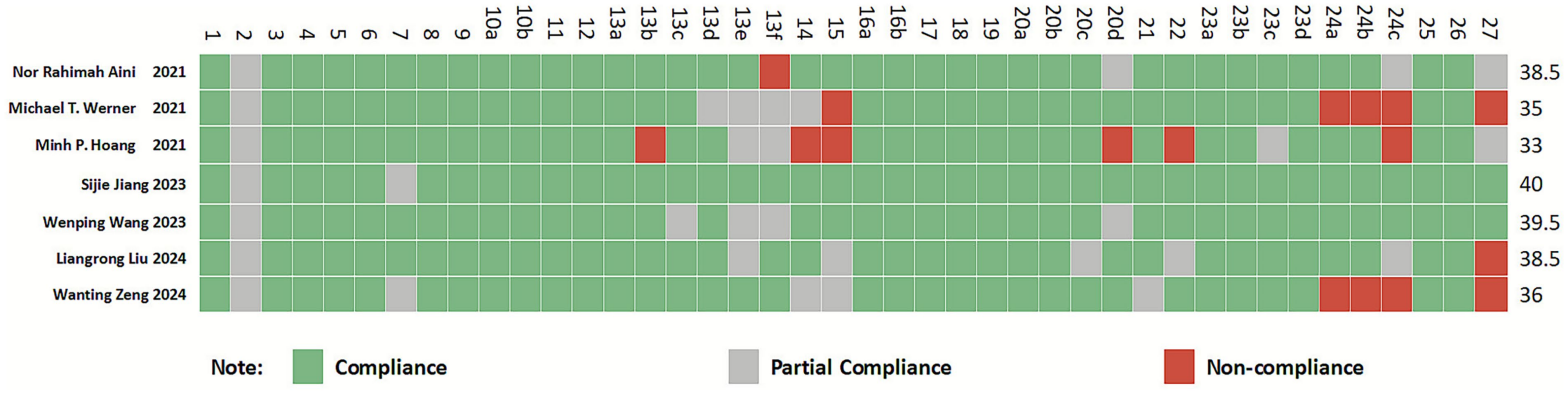
Cartesian heatmap of the scores of each item in PRISMA 2020.

All included studies (36–42) were rated as high quality. The PRISMA 2020 checklist consists of 27 items (42 sub-items), with a maximum score of 42 points. The included studies scored between 33 and 40 points, classified as “relatively complete reporting.” Among the 42 sub-items, items 2, 13e, 13f, 14, 15, 24c, and 27 showed lower reporting completeness across multiple studies, with significant information gaps. These deficiencies primarily involved sections such as the Abstract, Synthesis methods, Reporting bias assessment, Certainty assessment, Registration and protocol, and Availability of data, code, and other materials. For detailed information, refer to Figure 3.

#### 3.1.6 Results of the evidence quality assessment

The GRADE approach was used to assess the quality of evidence for the pooled outcome effect measures presented in forest plots across the included studies. A total of 64 pooled effect outcome measures were evaluated. The results demonstrated that 6 were rated as high-quality, 13 as moderate-quality, 18 as low-quality, and 27 as very low-quality. Detailed information is presented in Table 2.

**Table 2:** Evidence quality assessment.

The included studies	Endpoint measure	Downgrading factor					Effect size	95% CI	*I*^2^/%	*p*	Evidence quality
		RB	IC	ID	IP	PB					
Aini et al. (36)	CSMs	−1^①^	−1^②^	0	−1^④^	0	SMD = −0.51	[−1.31, 0.28]	71	0.210	Extremely low
	Symptoms score	0	−1^②^	0	−1^④^	0	SMD = −0.27	[−0.91, 0.38]	43	0.420	Low
	Medication score	0	−1^②^	0	−1^④^	0	SMD = −6.56	[−21.48, 8.37]	97	0.390	Low
	Rescue medication	0	0	0	−1^④^	0	RR = 12.32	[0.72, 211.79]	N/A	0.080	Medium
	Overall improvement score	0	−1^②^	0	−1^④^	0	MD = −0.07	[−2.28, 2.14]	75	0.950	Low
	Local swelling	0	−1^②^	0	−1^④^	0	RR = 4.51	[0.81, 25.06]	80	0.090	Low
	Abdominal symptoms	0	0	0	−1^④^	0	RR = 1.28	[0.24, 6.91]	0	0.780	Medium
	Nonspecific symptoms	0	0	0	0	0	RR = 1.19	[0.37, 3.79]	11	0.770	High
	Eye and nasal symptoms	0	−1^②^	0	0	0	RR = 1.00	[0.38, 2.59]	35	1.000	Medium
	Skin prick test	−1^①^	0	0	−1^④^	0	MD = −0.88	[−1.53, −0.23]	0	0.008	Low
	Specific IgE levels	0	0	0	−1^④^	0	MD = 5.63	[0.71, 10.55]	0	0.020	Medium
	Quality of life	0	−1^②^	0	0	0	SMD = −0.10	[−0.86, 0.67]	69	0.800	Medium
Werner and Bosso (37)	CSMs	−1^①^	0	0	0	−1^⑤^	SMD = −0.72	[−0.98, 0.46]	20.6	<0.0001	Medium
	Nasal provocation testing	−1^②^	0	0	0	0	SMD = −1.00	[−1.38, −0.61]	21.9	<0.0001	Low
	Skin prick testing	−1^②^	0	0	0	0	SMD = −0.73	[−0.99, −0.47]	5.3	<0.0001	Low
	Overall adverse event rate	−1^②^	−1^②^	0	−1^④^	−1^⑤^	RR = 2.20	[1.35, 3.59]	79.1	0.0015	Extremely low
Hoang et al. (38)	CSMs	−1^①^	0	0	−1^④^	0	SMD = −0.51	[−0.88, −0.14]	24	<0.01	Low
	VAS	−1^①^	0	0	0	0	MD = 2.98	[−0.96, 5.00]	0	<0.01	Medium
	Disease-specific quality of life	−1^①^	−1^②^	0	−1^④^	0	SMD = −0.40	[−1.21, 0.42]	N/A	0.34	Extremely low
	Specific IgG4 level	−1^①^	−1^②^	0	−1^④^	0	SMD = 0.70	[0.16, 1.24]	48	0.01	Extremely low
	Specific IgE level	−1^①^	0	0	0	0	SMD = 0.01	[−0.27, 0.30]	0	0.92	Medium
	Local reactions of adverse events	−1^①^	0	0	−1^④^	0	RR = 7.53	[1.82, 31.15]	N/A	0.01	Low
	Systemic reactions of adverse events	−1^①^	0	0	−1^④^	0	RR = 0.28	[0.12, 0.64]	N/A	<0.01	Low
	Symptom score	−1^①^	0	0	−1^④^	0	SMD = −0.28	[−0.74, 0.18]	0	0.23	Low
	Medication score	−1^①^	0	0	−1^④^	0	SMD = −0.14	[−0.16, 0.29]	0	0.48	Low
Jiang et al. (39)	CSMs	−1^①^	−1^②^	0	−1^④^	0	SMD = −0.85	[−1.58, −0.11]	85	0.22	Extremely low
	Allergic rhinoconjunctivitis quality of life	0	0	0	0	0	MD = −0.42	[−0.69, −0.15]	34	0.003	High
	VAS	−1^①^	−1^②^	0	−1^④^	0	MD = 0.60	[−1.16, 2.36]	86	0.50	Extremely low
	The impact of booster injection on CSMs	−1^①^	−1^②^	0	0	0	SMD = −4.81	[−6.54, 2.36]	N/A	<0.0001	Low
	The impact of no booster injection on CSMs	−1^①^	−1^②^	0	−1^④^	−1^⑤^	SMD = −0.76	[−1.43, −0.10]	N/A	0.03	Extremely low
	The impact of 4-week injection interval on CSMs	−1^①^	−1^②^	0	−1^④^	0	SMD = −1.00	[−1.86, −0.13]	N/A	0.02	Extremely low
	The impact of 2-week injection interval on CSMs	−1^①^	−1^②^	0	−1^④^	0	SMD = −0.01	[−0.68, −0.66]	N/A	0.97	Extremely low
	The impact of 4-week injection interval on VAS	−1^①^	0	0	0	0	MD = 1.25	[0.08, 2.42]	N/A	0.04	Medium
	The impact of 2-week injection interval on VAS	−1^①^	−1^②^	0	−1^④^	−1^⑤^	MD = −1.71	[−2.10, −1.32]	N/A	<0.0001	Extremely low
	Skin prick test	0	0	0	0	0	MD = −0.51	[−1.06, 0.04]	0	0.07	High
	Adverse events	−1^①^	−1^②^	0	−1^④^	0	RD = 0.16	[0.05, 0.27]	90	0.005	Extremely low
Wang et al. (40)	Local adverse events	0	−1^②^	0	−1^④^	0	RR = 2.99	[1.47, 6.07]	56	<0.05	Low
	Systemic adverse events	0	−1^②^	0	−1^④^	0	RR = 0.87	[0.62, 1.22]	56	0.43	Low
	Proportion of subjects experiencing adverse events	0	0	0	0	0	RR = 0.26	[0.12, 0.55]	0	<0.05	High
	Compliance	−1^①^	−1^②^	0	0	0	RR = 1.35	[0.91, 2.01]	91	0.13	Low
	CSMs	0	−1^②^	−1^③^	0	0	SMD = −0.37	[−0.68, −0.07]	0	0.02	Low
	Seasonal allergic rhinoconjunctivitis	0	0	0	−1^④^	0	SMD = −0.40	[−0.73, −0.07]	11	0.02	Medium
	4-Week Interval	0	0	0	0	0	SMD = −0.44	[−0.79, −0.10]	1	0.01	High
	Same doses	0	0	0	0	0	SMD = −0.46	[−0.82, −0.10]	0	0.01	High
	Escalating doses	0	−1^②^	0	−1^④^	0	SMD = −0.15	[−0.73, 0.43]	44	0.61	Low
Liu et al. (41)	Symptom score	−1^①^	0	−1^③^	−1^④^	0	SMD = 0.14	[−0.34, 0.62]	50.5	0.088	Extremely low
	Medication score	−1^①^	−1^②^	−1^③^	−1^④^	0	SMD = 1.37	[−0.45, 3.18]	92.3	0.001	Extremely low
	CSMs	−1^①^	−1^②^	−1^③^	−1^④^	0	SMD = 0.93	[−0.62, 2.47]	90.5	0.001	Extremely low
	Nasal symptoms	0	0	−1^③^	0	0	RR = 0.16	[0.06, 0.45]	0	0.995	Medium
	Lymphadenopathy	0	0	−1^③^	−1^④^	0	RR = 2.27	[0.37, 6.73]	49.3	0.116	Low
	Quality of life	−1^①^	0	−1^③^	0	0	SMD = −0.53	[−1.00, −0.050]	53	0.075	Low
	IgE	−1^①^	0	−1^③^	−1^④^	0	SMD = 0.93	[−0.44, 2.30]	50.5	0.001	Extremely low
Zeng et al. (42)	Symptom score	−1^①^	0	−1^③^	−1^④^	0	SMD = −0.20	[−0.62, 0.22]	4	0.35	Extremely low
	Medication score	−1^①^	0	−1^③^	−1^④^	0	SMD = −0.03	[−0.53, 0.48]	0	0.91	Extremely low
	CSMs	−1^①^	−1^②^	−1^③^	−1^④^	0	SMD = −0.28	[−0.90, 0.35]	60	0.39	Extremely low
	VAS	−1^①^	0	−1^③^	−1^④^	0	MD = 1.29	[0.60, 1.98]	0	0.0002	Extremely low
	Quality of life	−1^①^	−1^②^	−1^③^	−1^④^	0	SMD = 0.05	[−0.83, 0.92]	78	0.92	Extremely low
	The short-term efficacy of serum specific IgE	−1^①^	0	−1^③^	−1^④^	0	SMD = 0.03	[−0.40, 0.47]	0	0.88	Extremely low
	The medium-term efficacy of serum specific IgE	−1^①^	0	−1^③^	−1^④^	0	SMD = −0.04	[−0.37, 0.29]	0	0.80	Extremely low
	The long-term efficacy of serum specific IgE	−1^①^	0	−1^③^	−1^④^	0	SMD = 0.39	[−0.31, 1.09]	N/A	0.28	Extremely low
	The short-term efficacy of serum specific IgG4	−1^①^	0	−1^③^	−1^④^	0	SMD = 0.17	[−0.50, 0.84]	N/A	0.63	Extremely low
	The medium-term efficacy of serum specific IgG4	−1^①^	0	−1^③^	−1^④^	0	SMD = 0.35	[−0.06, 0.76]	0	0.10	Extremely low
	The long-term efficacy of serum specific IgG4	−1^①^	0	−1^③^	−1^④^	0	SMD = 0.12	[−0.57, 0.81]	N/A	0.74	Extremely low
	Skin prick test	−1^①^	0	−1^③^	−1^④^	0	MD = −0.88	[−1.53, −0.23]	N/A	0.008	Extremely low

RB, risk of bias; IC, inconsistency; ID, indirectness; IP, impression; PB, publication bias. ①Methodological quality of included studies was low, with biases in randomization, allocation concealment, and blinding. ②The heterogeneity was large and low confidence interval overlap. ③The population was not broadly representative. ④Small sample size, 95% confidence intervals include null values. ⑤Few studies were included, the funnel plot was not symmetrical, Egger’s test found that publication bias or results were positive, and there was no publication bias evaluation.

### 3.2 Quantitative analysis

#### 3.2.1 Combined symptom and medication score

**Figure 4:**
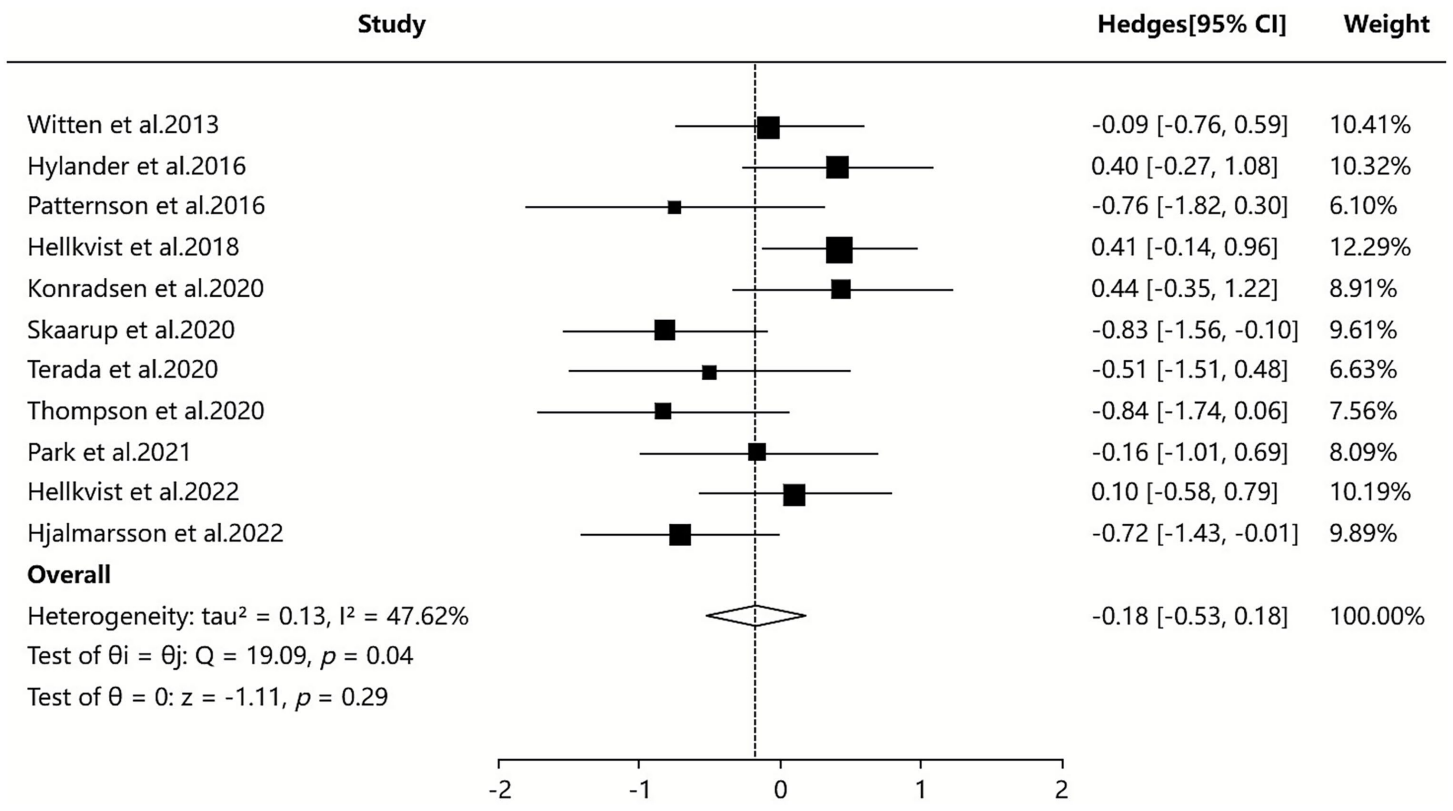
Meta-analysis of combined symptom and medication score.

The 7 SAs/MAs included in this study (36–42) all reported quantitative data on CSMs. Quantitative analysis using a random-effects model demonstrated that ILIT showed a statistically significant but modest effect on CSMs compared with placebo [SMD = −0.18, 95% CI (−0.53, 0.18), *p* = 0.04], with moderate heterogeneity observed among studies (*I*^2^ = 47.62%). Although sensitivity analyses and subgroup analyses were performed, the source of heterogeneity remained unclear. The results suggest that ILIT may provide slightly superior improvement in CSMs compared to placebo, as detailed in Figure 4.

#### 3.2.2 Symptom score

**Figure 5:**
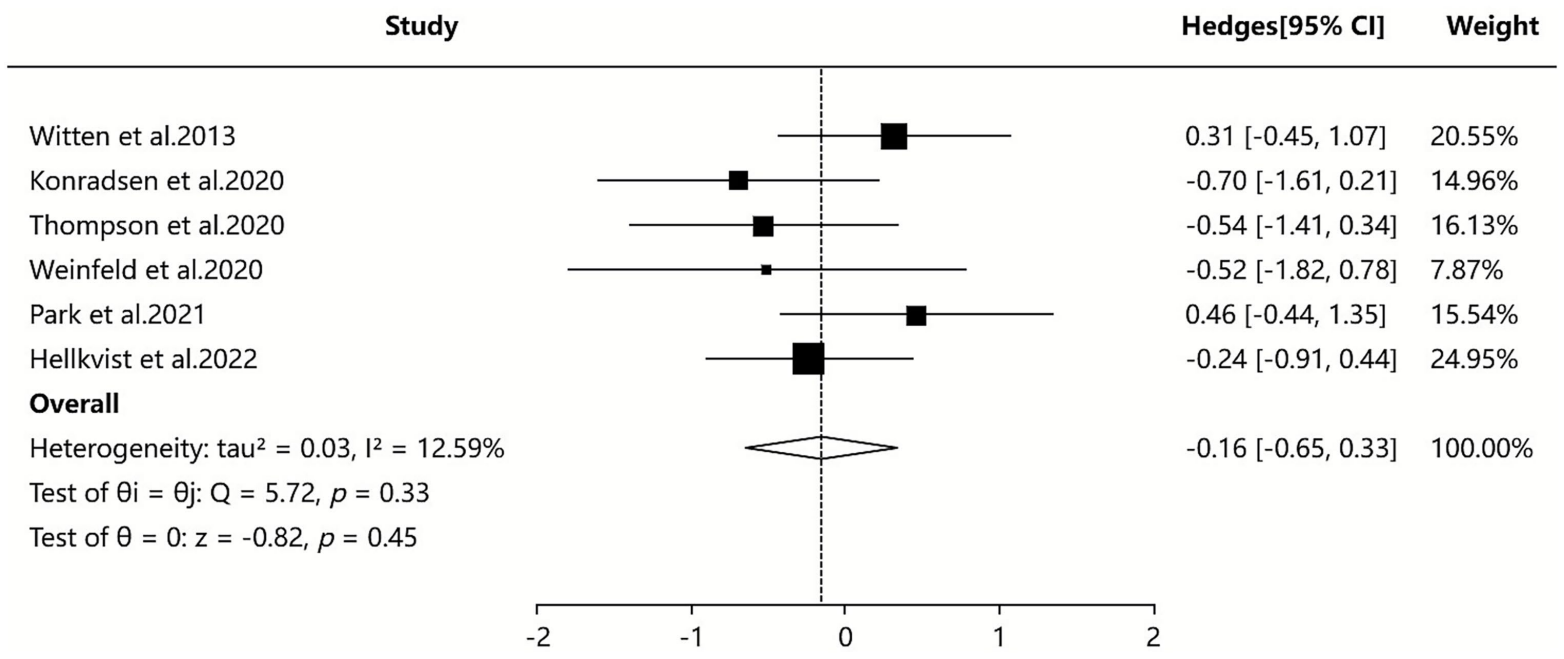
Meta-analysis of symptom score.

Three of the included SAs/MAs (36, 41, 42) reported quantitative data on symptom score. Quantitative analysis using a random-effects model showed that ILIT had a statistically significant but minimal effect on symptom scores compared to placebo [SMD = −0.16, 95% CI (−0.65, 0.33), *I*^2^ = 12.59%, *p* = 0.04]. The results indicate that symptom scores were slightly lower in the ILIT group than in the placebo group, but this difference did not demonstrate a statistically significant advantage. Details are presented in Figure 5.

#### 3.2.3 Serum-specific IgE

**Figure 6:**
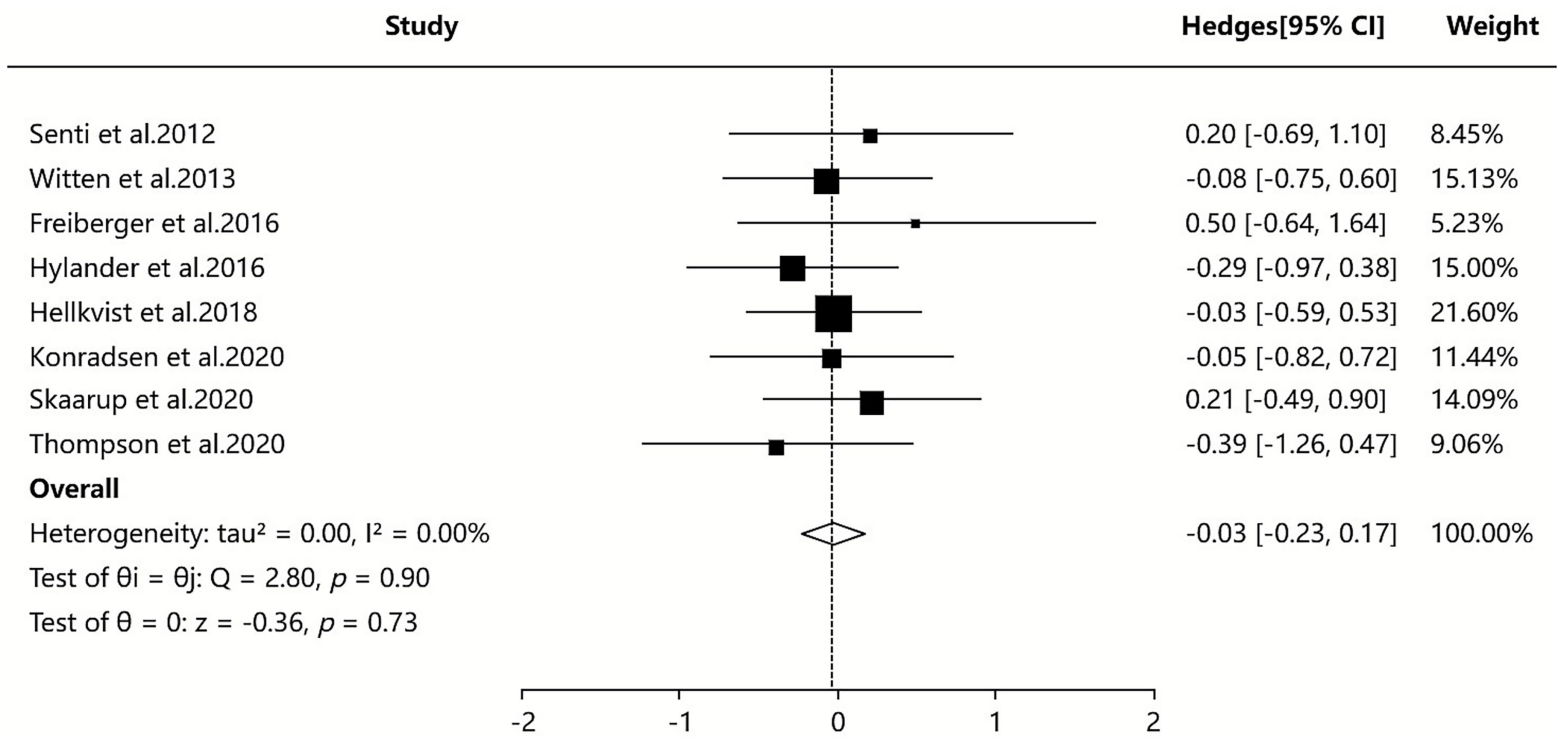
Meta-analysis of serum-specific IgE.

Three of the included SAs/MAs (38, 41, 42) reported quantitative data on serum IgE levels. Quantitative analysis using a random-effects model demonstrated that ILIT resulted in a marginally lower serum IgE level compared to placebo [SMD = −0.03, 95% CI (−0.20, 0.47), *I*^2^ = 0%, *p* = 0.90]. The findings indicate that ILIT did not show a statistically significant difference in reducing serum IgE levels in patients with AR. Further details are presented in Figure 6.

#### 3.2.4 Serum-specific IgG4

**Figure 7:**
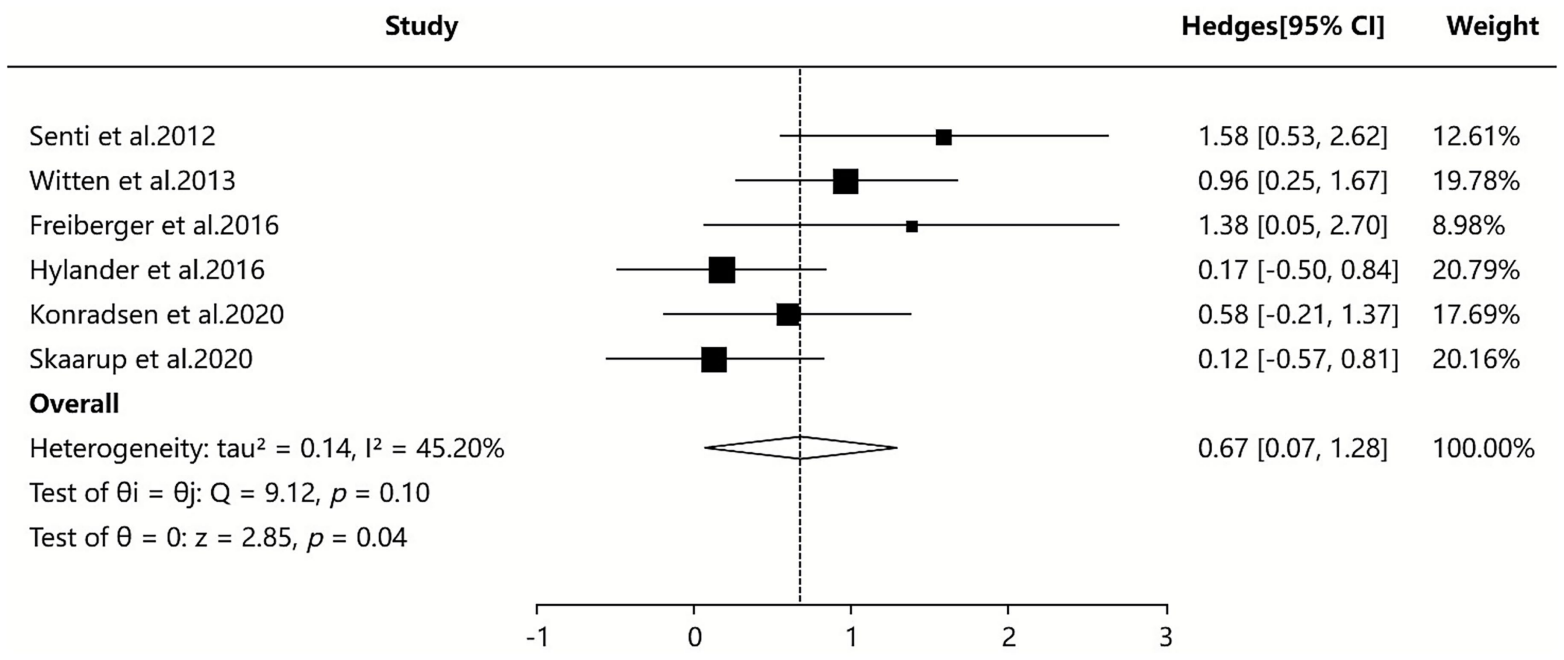
Meta-analysis of serum-specific IgG4.

Two SAs/MAs (38, 42) included in our study reported quantitative data on serum IgG4 levels. The quantitative analysis using a random-effects model indicated that the ILIT group showed higher serum IgG4 levels compared to the placebo group [SMD = 0.67, 95% CI (0.07–1.28), *p* = 0.10], with moderate between-study heterogeneity (*I*^2^ = 45.20%). Although sensitivity and subgroup analyses were performed, the source of heterogeneity could not be definitively identified. The results suggest that ILIT demonstrated a trend toward increasing serum IgG4 levels in patients with AR compared to placebo (albeit without reaching statistical significance). This finding may indirectly indicate that ILIT successfully induces key immune tolerance mechanisms. Detailed results are presented in Figure 7.

### 3.3 Qualitative analysis

#### 3.3.1 Adverse even or adverse reaction

Among the included studies, six SAs/MAs (36–40, 42) used adverse event or adverse reaction as outcome measures. All six articles indicated that the incidence of local adverse reactions was significantly higher with ILIT than with placebo, but there was no statistically significant difference in systemic adverse reactions between ILIT and placebo, with most systemic reactions being mild. These six studies all emphasized that ILIT had a controllable safety profile, and no fatal adverse events were reported. Two SAs/MAs (36, 40) suggested that high-dose ILIT might lead to severe allergic reactions and should be used with caution. Two other SAs/MAs (39, 42) compared the safety of ILIT and SCIT, finding that ILIT caused more local reactions but significantly fewer systemic reactions than SCIT. Additionally, two SAs/MAs (37, 39) noted that a few studies reported no new severe adverse events during long-term follow-up, though most studies lacked long-term safety data.

#### 3.3.2 Quality of life

Among the included studies, six SAs/MAs (36–39, 41, 42) assessed quality of life as an outcome measure. Five SAs/MAs (36–38, 41, 42) reported no significant difference in quality of life scores between the ILIT and placebo groups, indicating that ILIT did not significantly improve quality of life in patients with AR. Only one SAs/MAs (39) found a slight improvement in quality of life with ILIT. Four SAs/MAs (36, 38, 41, 42) observed that ILIT did not significantly enhance the quality of life in the short term (<24 weeks of follow-up), though long-term follow-up data were lacking. Three SAs/MAs (36, 39, 42) focusing specifically on grass pollen-allergic patients reported no significant the quality of life improvement with ILIT. However, studies involving multiple allergens (e.g., house dust mites, pet dander) suggested variable effects, with one study (41) indicating that ILIT might be more effective in improving the quality of life for patients sensitized to multiple allergens.

#### 3.3.3 Medication score

Four SAs/MAs (36, 38, 41, 42) demonstrated that ILIT showed no significant advantage over placebo in terms of medication score improvement, indicating its limited effect on ameliorating medication scores in AR patients. However, one SAs/MAs (37) presented discordant findings, suggesting that ILIT had significant therapeutic efficacy. Regarding injection intervals, two SAs/MAs (37, 38) proposed that a 4-week injection interval might yield better improvement in medication scores, while another two studies (36, 41) found no significant influence of injection interval on medication scores.

#### 3.3.4 Visual analog scale

Four SAs/MAs (37, 38, 40, 42) indicated that ILIT demonstrated significant efficacy in improving the VAS, particularly in the short term (<24 weeks) and medium term (24–52 weeks). However, two SAs/MAs (38, 40) reported that ILIT showed no significant long-term (>52 weeks) effect on the VAS improvement, while also finding comparable efficacy between ILIT and SCIT in terms of the VAS outcomes. Additionally, two SAs/MAs (38, 39) revealed that the injection interval of ILIT influenced its effect on the VAS, with a 4-week interval demonstrating superior efficacy compared to a 2-week interval. Furthermore, two SAs/MAs (40, 42) found that ILIT was more effective in improving the VAS in seasonal AR than in perennial AR.

#### 3.3.5 Skin prick test

Three SAs/MAs (36, 39, 42) demonstrated that ILIT could improve skin prick test results by reducing wheal diameter, indicating a decreased skin reaction to allergens. Two SAs/MAs (36, 37) suggested that the efficacy of ILIT on skin prick test outcomes was comparable to that of conventional immunotherapy. Additionally, two SAs/MAs (36, 42) reported that the effects of ILIT on skin prick test were dose- and injection interval-dependent.

#### 3.3.6 Overall/subjective improvement

Among the included studies, two SAs/MAs (36, 40) used Overall/subjective improvement as outcome measures. However, the conclusions were inconsistent. The study by Aini et al. (36) demonstrated no significant difference in overall improvement scores between ILIT and placebo, whereas the study by Wang et al. (40) reported that the subjective improvement score of ILIT was significantly lower than that of placebo, with this effect being more pronounced in seasonal AR.

#### 3.3.7 Other outcome measures

Three SAs/MAs (37, 40, 42) focused on nasal provocation tests and reached consistent conclusions, demonstrating that ILIT improves nasal provocation test outcomes. ILIT effectively enhances patients’ tolerance to allergens and reduces nasal symptoms. The study by Aini et al. (36) found no significant difference between ILIT and placebo in reducing the use of rescue medications. In contrast, Werner et al. (37) reported that ILIT resulted in significantly lower conjunctival provocation test responses compared to placebo, indicating a notable improvement in conjunctival provocation test outcomes. Additionally, Wang et al. (40) revealed that AR patients receiving ILIT exhibited higher treatment compliance. Liu et al. (41) further demonstrated that ILIT significantly reduces nasal symptoms with considerable clinical efficacy, while not significantly increasing the risk of lymphadenopathy.

## 4 Discussion

### 4.1 The efficacy and safety of ILIT in the treatment of allergic rhinitis

SIT, a treatment modality that combats allergic diseases by activating or enhancing the human immune system (11, 43), has been clinically applied for decades. As an innovative approach to SIT, ILIT has recently garnered significant attention. Unlike traditional methods such as SCIT (44) or SLIT (45), ILIT utilizes ultrasound guidance (46) to deliver allergen extracts directly into superficial lymph nodes, establishing a more targeted and efficient immune modulation pathway (47). This study demonstrates that ILIT exhibits moderate efficacy in improving symptoms and certain objective parameters in AR patients, though its effects are influenced by treatment duration, allergen type, and dosing regimens. While short- and medium-term outcomes (≤52 weeks) are favorable, long-term efficacy (>52 weeks) appears limited. Notably, ILIT confers significant clinical benefits in AR induced by grass pollen or mixed allergens, but its therapeutic impact is less pronounced in single-house dust mite -mediated cases. Furthermore, an incremental dosing protocol or a 4-week interval injection strategy significantly enhances the dose–response relationship of ILIT.

Allergen immunotherapy is inherently an artificial intervention in the immune system, and safety serves as the bottom line of allergen immunotherapy (48–50). Its importance permeates the entire chain of treatment feasibility, patient acceptance, and clinical promotion. This study found that the overall safety profile of ILIT remains within a controllable range, with adverse events primarily manifesting as mild local reactions. The risk of systemic adverse reactions is relatively low and mostly mild, with no reports of fatal events to date. It is noteworthy that high-dose regimens may increase the risk of severe anaphylaxis and thus require cautious application. Compared to SCIT, ILIT is associated with a higher incidence of local reactions but significantly fewer systemic reactions. Regarding long-term safety, available data are limited. A few studies suggest that no new severe adverse events were observed during long-term follow-up; however, most studies lack extended tracking data. From the patient perspective, adherence to ILIT is generally high.

Our findings are consistent with the core conclusions of previous SAs/MAs on ILIT for AR, while addressing critical gaps in standardized efficacy comparisons, long-term therapeutic mechanisms, safety stratification, and subgroup response patterns. However, the reevaluation of SAs/MAs on ILIT for AR should extend beyond simplistic “effective/ineffective” or “safe/unsafe” dichotomous conclusions. Through this investigation, we have identified several novel insights of unique value: (1) Distinct from the gradual immunomodulation of conventional immunotherapy (SCIT/SLIT), ILIT employs pulsed administration to trigger an “immunological memory burst window” - characterized by a rapid surge in the IgG4/IgE ratio at specific time points, followed by a plateau phase; (2) Anatomically Targeted Immunomodulation of Lymphatic Microenvironments: Dose requirements may exhibit regional heterogeneity across lymphatic sites. Cervical lymph nodes demonstrate superior efficiency in capturing antigen-presenting cells derived from nasal mucosa, while inguinal lymph nodes are more suitable for systemic allergen immunotherapy; (3) The Immunomodulation Time Window of ILIT: Building upon established principles of immune tolerance induction (51), ILIT activates lymph node-resident dendritic cells to initiate antigen-specific regulatory T cell differentiation. Consequently, excessively short dosing intervals may lead to immune cell exhaustion due to frequent antigen exposure, while prolonged intervals could compromise sustained immune activation; (4) Cost-Effectiveness Adaptability of ILIT: While ILIT incurs lower direct costs than SCIT/SLIT, its expense remains higher than conventional therapies. However, when accounting for reduced productivity loss among patients and caregivers, ILIT demonstrates superior overall cost-efficiency. From a socioeconomic perspective, this approach simultaneously alleviates family burdens, enhances societal productivity, and addresses the needs of medically underserved regions, representing an economically viable and practical therapeutic option.

### 4.2 Evaluation of literature quality

All seven SAs/MAs included in this study were rigorously assessed using the ROBIS tool, with all meeting the low risk of bias criteria, further reinforcing the reliability of our findings. Future research should ensure comprehensive consideration during the study design phase, including clearly predefined inclusion criteria. Literature searches must be exhaustive to avoid omitting pivotal studies. Data extraction and quality assessment should adhere to strict methodological standards, while data synthesis and result presentation must be appropriate. Additionally, potential biases at each stage should be carefully addressed when interpreting the results.

The AMSTAR-2 assessment revealed that among the SAs/MAs included in this study, 6 were rated as low quality and one as critically low quality. The primary limitations were as follows: (1) Failure to provide a list of excluded studies along with justification for exclusion, which compromises transparency in the selection process, hinders the evaluation of screening rationality, and may lead to omission of critical information, thereby increasing potential bias and significantly undermining the reliability and persuasiveness of the results. (2) Lack of reporting on funding sources of the included studies, preventing assessment of potential conflicts of interest and sponsorship bias, which diminishes the credibility of the findings and impedes accurate judgment of the objectivity and generalizability of the conclusions. (3) The failure to assess the risk of bias in individual studies for its potential impact on meta-analysis results or other evidence syntheses renders the findings lacking in consideration of bias interference. This makes it difficult to evaluate the true contribution of each study’s results to the overall conclusion and hampers the effective identification and control of biases. (4) Failure to consider the risk of bias in original studies may lead to inaccurate assessment of the credibility of research findings, difficulty in identifying whether the results are exaggerated or underestimated due to bias, inability to reveal potential flaws in the study, and reduced reliability and scientific rigor of the conclusions.

According to the PRISMA 2020 evaluation, most studies neglect to assess the certainty of the evidence system, fail to disclose protocol registration information and amendment records, and lack accessibility statements for data codes and related materials. These deficiencies lead to fragmentation in result synthesis and application logic, thereby creating blind spots in research design standardization and bias risk assessment. In conclusion, future SAs/Mas should prospectively plan the evaluation of evidence certainty, standardize the disclosure of protocol registration information, amendment records, and data code accessibility, and enhance research transparency and traceability through standardized procedures.

In the GRADE system ratings, many included studies were downgraded due to risks of bias in key methodological aspects such as randomization, allocation concealment, and blinding. Additionally, numerous studies were downgraded because of substantial heterogeneity, low overlap of confidence intervals, and small sample sizes, leading to insufficient evidence consistency and precision. Therefore, it is recommended that future SAs/MAs enhance the methodological rigor of primary studies by strictly implementing randomization and allocation concealment, pre-specifying heterogeneity analysis plans, and optimizing heterogeneity management. Furthermore, future primary studies should perform *a priori* sample size calculations to ensure adequate statistical power.

### 4.3 Current research controversies and future research suggestions

In the course of this SAs/MAs, the evaluation results of ILIT efficacy exhibited multi-dimensional discrepancies and controversies. (1) Symptom scores: The study by Aini et al. (36) showed differences compared to four other studies, which may be attributed to variations in study design or heterogeneity handling methods. (2) Medication scores: The impact of ILIT on medication scores and the contradictory conclusions regarding injection intervals may be related to the specific allergens included or differences in scoring methodologies. (3) Quality of life: The study by Jiang et al. (39) reported only slight improvements, a finding that might be constrained by limited follow-up duration, while subjective patient perceptions could also introduce bias in the assessment. (4) Overall improvement: Two studies (36, 40) yielded divergent conclusions, likely due to differences in study population characteristics and variations in the sensitivity of assessment tools. (5) Serum-specific IgG4: The contradictory findings between two studies (38, 42) may stem from differences in treatment dosage, injection interval protocols, and the selection of time points for IgG4 measurement.

Based on the aforementioned inter-study discrepancies and the varying degrees of heterogeneity identified through quantitative analysis, we have identified several potential sources of heterogeneity, including differences in patient populations, variations in allergen types and dosages, inconsistencies in injection intervals and treatment duration, as well as disparities in assessment tools and definitions of outcome measures. To enhance the reliability and consistency of findings, we recommend that future trials adopt larger sample sizes and standardized ILIT protocols. Subsequent research should focus on optimizing study designs to minimize heterogeneity, exploring personalized regimens for dosage and administration intervals, extending follow-up durations to evaluate long-term safety and efficacy, strengthening mechanistic investigations and subgroup analyses, as well as conducting comparative studies with SCIT and exploring combination therapies. These efforts will help to clarify the clinical value of ILIT and provide a solid foundation for precision medicine in AR treatment.

### 4.4 Limitation

This study has several limitations: (1) Among the included SAs/MAs, it was impossible to control for potential biases in the original studies, which may have influenced the results to some extent. (2) The seven SAs/MAs included in this study exhibited high reporting quality but demonstrated suboptimal methodological quality. Furthermore, there was a non-negligible degree of overlap among the primary RCTs across these reviews, which may potentially affect the robustness and reliability of the evidence synthesis. (3) Given the generally low methodological quality of the included studies, the findings of this research should be interpreted with caution, and overinterpretation should be avoided. (4) The researchers’ inherent subjectivity during the study process may have introduced bias into the evaluation results.

## 5 Conclusion

Evidence from published SAs/MAs suggests that ILIT demonstrates clinically meaningful improvements in both symptom control and selected objective parameters for AR, with well-established short-to-medium term efficacy that appears more limited in long-term follow-up. The therapeutic benefits are particularly evident for grass pollen and mixed-allergen induced AR, while maintaining a favorable safety profile characterized predominantly by mild local reactions. Moving forward, ILIT requires transformative innovation to evolve from empirical administration to mechanism-driven precision immunomodulation. Based on current evidence, four critical research directions emerge: (1) Development of immunophenotype predictive models incorporating novel biomarkers ranging from IgG4/IgE ratios to IL-35 signatures; (2) Exploration of synergistic tolerance potential with biologicals, positioning ILIT as a bridging therapy in AR step-up protocols; (3) Quantitative analysis of technology-dependent efficacy through standardized assessment of ultrasound-guided precision and lymph node targeting accuracy; (4) Investigation of bidirectional “local-systemic immune axis” regulation, particularly ILIT-mediated remote remodeling of nasal mucosal microenvironments. Given the current scarcity of long-term efficacy and safety data on ILIT, it is recommended that future studies should involve large-sample, standardized, multi-center, long-term follow-up randomized controlled trials across different countries and regions, targeting diverse age groups and incorporating varied injection doses and intervals. Such trials would allow for a systematic evaluation of its sustained efficacy, delayed adverse reactions, and long-term immunomodulatory effects. Additionally, establishing a real-world data platform that integrates electronic health records and patient-reported outcomes could help address the limitations of clinical trials in terms of population representativeness and long-term observation, thereby providing more comprehensive evidence to support clinical decision-making.

## Data Availability

The original contributions presented in the study are included in the article/Supplementary material, further inquiries can be directed to the corresponding author.
