# Supplementary material for: Efficacy and safety of intralymphatic immunotherapy for allergic rhinitis: an overview of systematic reviews and meta analyses
Source: Front Med (Lausanne). 2025 Dec 5;12:1709531. doi: 10.3389/fmed.2025.1709531 (PMC12714667; doi:10.3389/fmed.2025.1709531)
Supplement: Supplementary file 1 [file Supplementary_file_1.doc]

Research Process and Research Data

Contents

[1.Search Query and Search Logic 2](#__RefHeading___Toc12639)

[2.ROBIS 5](#__RefHeading___Toc19775)

[3.AMSTAR-2](#__RefHeading___Toc31481) 8

[4.PRISMA 2020 1](#__RefHeading___Toc14575)5

[5.GRADE 4](#__RefHeading___Toc2063)5

[6.Excluded literature and the reasons for exclusion 5](#__RefHeading___Toc9305)4

1.Search Query and Search Logic

PubMed

(((((((((Rhinitis, Allergic[MeSH Terms]) OR (Rhinitis, Allergic[Title/Abstract])) OR (Allergic Rhinitis[Title/Abstract])) OR (hypersensitive rhinitis[Title/Abstract])) OR (anaphylactic rhinitis[Title/Abstract])) OR (nasal allergy[Title/Abstract])) OR (rhinallergosis[Title/Abstract])) OR (Allergic rhinoconjunctivitis[Title/Abstract])) AND (((Intralymphatic immunotherapy[Title/Abstract]) OR (Intralymphatic immunization[Title/Abstract])))) AND (((((Meta-Analysis[Publication Type]) OR (Meta-Analysis[Title/Abstract])) OR (systematic review[Title/Abstract])) OR (systematic review[Publication Type])))

Cochrane Library

#1:MeSH descriptor: [Rhinitis, Allergic] explode all trees

#2:(allergic rhinitis):ti,ab,kw OR (rhinitis, allergic):ti,ab,kw OR (hypersensitive rhinitis):ti,ab,kw OR (anaphylactic rhinitis):ti,ab,kw OR (nasal allergy):ti,ab,kw OR (rhinallergosis):ti,ab,kw OR (allergic rhinoconjunctivitis):ti,ab,kw

#3:#1 OR #2

#4:(Intralymphatic immunotherapy):ti,ab,kw OR (Intralymphatic immunization):ti,ab,kw

#5:MeSH descriptor: [Meta-Analysis] explode all trees

#6:MeSH descriptor: [Systematic Review] explode all trees

#7:(meta):ti,ab,kw OR (meta analysis):ti,ab,kw OR (meta-analysis):ti,ab,kw OR (systematic review):ti,ab,kw

#8:#5 OR #6 OR #7

#9:#3 AND #4 AND #8

Embase

#1:'allergic rhinoconjunctivitis'/exp OR 'allergic rhinoconjunctivitis'

#2:'allergic rhinitis'/exp OR 'allergic rhinitis'

#3:'rhinitis, allergic'

#4:'hypersensitive rhinitis'

#5:'anaphylactic rhinitis'

#6:'nasal allergy'

#7:rhinallergosis

#8:#1 OR #2 OR #3 OR #4 OR #5 OR #6 OR #7

#9:'Intralymphatic immunotherapy'

#10:'Intralymphatic immunization'

#11:#9 OR #10

#12:'meta analysis'/exp OR 'meta analysis'

#13:'systematic review'/exp OR 'systematic review'

#14:#12 OR #13

#15:#8 AND #11 AND #14

Web of Science

#1:(((((TS=(allergic rhinitis)) OR TS=(rhinitis, allergic)) OR TS=(hypersensitive rhinitis)) OR TS=(anaphylactic rhinitis)) OR TS=(rhinallergosis)) OR TS=(allergic rhinoconjunctivitis)

#2:(TS=(Intralymphatic immunotherapy)) OR TS=(Intralymphatic immunization)

#3:(((TS=(meta analysis)) OR TS=(meta)) OR TS=(meta-analysis)) OR TS=(systematic review)

#4:#1 AND #2 AND #3

CNKI

(SU=变应性鼻炎 OR SU=过敏性鼻炎 OR SU=变应性鼻炎患儿 OR SU=过敏性鼻炎患儿 OR SU=变应性鼻炎患者 OR SU=过敏性鼻炎患者 OR SU=变应性鼻炎疗法 OR SU=过敏性鼻炎疗法 OR SU=变应性鼻炎治疗 OR SU=过敏性鼻炎治疗 OR SU=变应性鼻结膜炎 OR SU=过敏性鼻结膜炎) AND (SU=淋巴结内免疫 OR SU=淋巴结内注射免疫 OR SU=淋巴结内注射变应原特异性免疫 SU=淋巴结内特异性免疫 OR SU=淋巴结免疫 OR SU=淋巴结注射免疫 OR SU=淋巴结) AND (SU=meta OR SU=meta分析 OR SU=’meta-analysis’ OR SU=’meta analysis’ OR SU=荟萃分析 OR SU=元分析)

VIP

(M=变应性鼻炎 OR M=过敏性鼻炎 OR M=变应性鼻炎患儿 OR M=过敏性鼻炎患儿 OR M=变应性鼻炎患者 OR M=过敏性鼻炎患者 OR M=变应性鼻炎疗法 OR M=过敏性鼻炎疗法 OR M=变应性鼻炎治疗 OR M=过敏性鼻炎治疗 OR M=变应性鼻结膜炎 OR M=过敏性鼻结膜炎) AND (M=淋巴结内免疫 OR M=淋巴结内注射免疫 OR M=淋巴结内注射变应原特异性免疫 OR M=淋巴结内特异性免疫 OR M=淋巴结免疫 OR M=淋巴结注射免疫 OR M=淋巴结) AND (M=meta OR R=“meta分析” OR R= “meta-analysis” OR R=“meta analysis” OR R=荟萃分析 OR R=元分析 OR R=系统评价)

WANFANG

主题:(变应性鼻炎 or 过敏性鼻炎 or 变应性鼻炎患儿 or 过敏性鼻炎患儿 or 变应性鼻炎患者 or 过敏性鼻炎患者 or 变应性鼻炎疗法 or 过敏性鼻炎疗法 or 变应性鼻炎治疗 or 过敏性鼻炎治疗 or 变应性鼻结膜炎 or 过敏性鼻结膜炎) and 主题:(淋巴结内免疫 or 淋巴结内注射免疫 or 淋巴结内注射变应原特异性免疫 or 淋巴结内特异性免疫 or 淋巴结免疫 or 淋巴结注射免疫 or 淋巴结) and 主题:(meta or R“meta分析” or “meta-analysis” or “meta analysis” or 荟萃分析 or 元分析 or 系统评价)

CBM

 (("变应性鼻炎治疗"[标题:智能] OR "过敏性鼻结膜炎"[标题:智能] OR "变应性鼻结膜炎"[标题:智能]) OR ("过敏性鼻炎疗效"[标题:智能] OR "变应性鼻炎疗效"[标题:智能] OR "过敏性鼻炎"[标题:智能] OR "变应性鼻炎"[标题:智能] OR "变应性鼻炎患儿"[标题:智能] OR "变应性鼻炎患者"[标题:智能] OR "过敏性鼻炎患儿"[标题:智能] OR "过敏性鼻炎患者"[标题:智能] OR "过敏性鼻炎治疗"[标题:智能]) OR (("鼻炎, 过敏性"[不加权:扩展]) OR "鼻炎, 变应性, 季节性"[不加权:扩展])) AND ("淋巴结内免疫"[标题:智能] OR "淋巴结内注射免疫"[标题:智能] OR "淋巴结内注射变应原特异性免疫"[标题:智能] OR "淋巴结内特异性免疫"[标题:智能] OR "淋巴结免疫"[标题:智能] OR "淋巴结注射免疫"[标题:智能] OR "淋巴结"[标题:智能]) AND (("meta"[标题:智能] OR "meta分析"[标题:智能] OR "meta-analysis"[标题:智能] OR "meta"[标题:智能] AND "analysis"[标题:智能] OR "荟萃分析"[标题:智能] OR "元分析"[标题:智能] OR "系统评价"[标题:智能]) OR (("系统评价(主题)"[不加权:扩展]) OR "Meta分析"[不加权:扩展] OR "Meta分析(主题)"[不加权:扩展]))

2.ROBIS

P: Pass. F: Fail. L: Low risk of bias. H: High risk of bias. Un: Unclear risk of bias.

A:The conclusions of researcher Ph.D. Zhuang Wang.

B:The conclusions of researcher Ph.D. Xiaofei Xie.

C:In case of a difference of opinions, it shall be adjudicated by Professor Zhikai Qiu.

D:Conclusive conclusion.

Study 1：Aini NR, Mohd Noor N, Md Daud MK, Wise SK, Abdullah B. Efficacy and safety of intralymphatic immunotherapy in allergic rhinitis: A systematic review and meta-analysis. Clin Transl Allergy. 2021 Aug 17;11(6):e12055. doi: 10.1002/clt2.12055. PMID: 34429875; PMCID: PMC8369948.

| Phase | | A | B | C | D |
| --- | --- | --- | --- | --- | --- |
| Phase 1: Assessing Relevance | | P | P | - | P |
| Phase 2: Identifying Concerns with Review Process | Study Eligibility Criteria | L | L | - | L |
|  | Identification and Selection of Studies | L | L | - | L |
|  | Data Collection and Study Appraisal | L | L | - | L |
|  | Synthesis and Findings | L | L | - | L |
| Phase 3: Judging Risk of Bias | | L | L | - | L |

Study 2：Werner MT, Bosso JV. Intralymphatic immunotherapy for allergic rhinitis: A systematic review and meta-analysis. Allergy Asthma Proc. 2021 Jul 1;42(4):283-292. doi: 10.2500/aap.2021.42.210028. PMID: 34187620.

| Phase | | A | B | C | D |
| --- | --- | --- | --- | --- | --- |
| Phase 1: Assessing Relevance | | P | P | - | P |
| Phase 2: Identifying Concerns with Review Process | Study Eligibility Criteria | L | L | - | L |
|  | Identification and Selection of Studies | L | L | - | L |
|  | Data Collection and Study Appraisal | L | L | - | L |
|  | Synthesis and Findings | L | L | - | L |
| Phase 3: Judging Risk of Bias | | L | L | - | L |

Study3：Hoang MP, Seresirikachorn K, Chitsuthipakorn W, Snidvongs K. Intralymphatic immunotherapy for allergic rhinoconjunctivitis: a systematic review and meta-analysis. Rhinology. 2021 Jun 1;59(3):236-244. doi: 10.4193/Rhin20.572. PMID: 33647073.

| Phase | | A | B | C | D |
| --- | --- | --- | --- | --- | --- |
| Phase 1: Assessing Relevance | | P | P | - | P |
| Phase 2: Identifying Concerns with Review Process | Study Eligibility Criteria | L | L | - | L |
|  | Identification and Selection of Studies | L | L | - | L |
|  | Data Collection and Study Appraisal | L | L | - | L |
|  | Synthesis and Findings | L | L | - | L |
| Phase 3: Judging Risk of Bias | | L | L | - | L |

Study 4：Jiang S, Xie S, Tang Q, Zhang H, Xie Z, Zhang J, Jiang W. Evaluation of Intralymphatic Immunotherapy in Allergic Rhinitis Patients: A Systematic Review and Meta-analysis. Mediators Inflamm. 2023 May 8;2023.

| Phase | | A | B | C | D |
| --- | --- | --- | --- | --- | --- |
| Phase 1: Assessing Relevance | | P | P | - | P |
| Phase 2: Identifying Concerns with Review Process | Study Eligibility Criteria | L | L | - | L |
|  | Identification and Selection of Studies | L | L | - | L |
|  | Data Collection and Study Appraisal | L | L | - | L |
|  | Synthesis and Findings | L | L | - | L |
| Phase 3: Judging Risk of Bias | | L | L | - | L |

Study 5：Wang W, Wang X, Wang H, Wang X. Evaluation of Safety, Efficacy, and Compliance of Intralymphatic Immunotherapy for Allergic Rhinoconjunctivitis: A Systematic Review and Meta-Analysis. Int Arch Allergy Immunol. 2023;184(8):754-766. doi: 10.1159/000529025. Epub 2023 Apr 27. PMID: 37105134.

| Phase | | A | B | C | D |
| --- | --- | --- | --- | --- | --- |
| Phase 1: Assessing Relevance | | P | P | - | P |
| Phase 2: Identifying Concerns with Review Process | Study Eligibility Criteria | L | L | - | L |
|  | Identification and Selection of Studies | L | L | - | L |
|  | Data Collection and Study Appraisal | L | L | - | L |
|  | Synthesis and Findings | L | L | - | L |
| Phase 3: Judging Risk of Bias | | L | L | - | L |

Study 6：Liu L, Liang Y, Yan L, Li Z. Is intralymphatic immunotherapy effective and safe for allergic rhinitis?: A meta-analysis. Medicine (Baltimore). 2024 Nov 15;103(46):e40589. doi: 10.1097/MD.0000000000040589. PMID: 39560522; PMCID: PMC11576000.

| Phase | | A | B | C | D |
| --- | --- | --- | --- | --- | --- |
| Phase 1: Assessing Relevance | | P | P | - | P |
| Phase 2: Identifying Concerns with Review Process | Study Eligibility Criteria | L | L | - | L |
|  | Identification and Selection of Studies | L | L | - | L |
|  | Data Collection and Study Appraisal | L | L | - | L |
|  | Synthesis and Findings | L | L | - | L |
| Phase 3: Judging Risk of Bias | | L | L | - | L |

Study 7：曾婉婷,李艮平,蒋建国,等.淋巴结内特异性免疫治疗变应性鼻炎疗效和安全性的Meta分析[J].中国耳鼻咽喉颅底外科杂志,2024,30(02):9-18.

| Phase | | A | B | C | D |
| --- | --- | --- | --- | --- | --- |
| Phase 1: Assessing Relevance | | P | P | - | P |
| Phase 2: Identifying Concerns with Review Process | Study Eligibility Criteria | L | L | - | L |
|  | Identification and Selection of Studies | L | L | - | L |
|  | Data Collection and Study Appraisal | L | L | - | L |
|  | Synthesis and Findings | L | L | - | L |
| Phase 3: Judging Risk of Bias | | L | L | - | L |

3.AMSTAR-2

Y: Yes; N: No; PY: Partial Yes.

A:The conclusions of researcher Ph.D. Zhuang Wang.

B:The conclusions of researcher Ph.D. Xiaofei Xie.

C:In case of a difference of opinions, it shall be adjudicated by Professor Zhikai Qiu.

D:Conclusive conclusion.

Study 1：Aini NR, Mohd Noor N, Md Daud MK, Wise SK, Abdullah B. Efficacy and safety of intralymphatic immunotherapy in allergic rhinitis: A systematic review and meta-analysis. Clin Transl Allergy. 2021 Aug 17;11(6):e12055. doi: 10.1002/clt2.12055. PMID: 34429875; PMCID: PMC8369948.

| Entry | | A | B | C | D |
| --- | --- | --- | --- | --- | --- |
| 1 | Did the research questions and inclusion criteria for the review include the components of PICO? | Y | Y | - | Y |
| 2 | Did the report of the review contain an explicit statement that the review methods were established prior to the conduct of the review and did the report justify any significantdeviations from the protocol? | Y | Y | - | Y |
| 3 | Did the review authors explain their selection of the study designs for inclusion in the review? | Y | Y | - | Y |
| 4 | Did the review authors use a comprehensive literature search strategy? | Y | Y | - | Y |
| 5 | Did the review authors perform study selection in duplicate? | Y | Y | - | Y |
| 6 | Did the review authors perform data extraction in duplicate? | PY | Y | Y | Y |
| 7 | Did the review authors provide a list of excluded studies and justify the exclusions? | PY | Y | PY | PY |
| 8 | Did the review authors describe the included studies in adequate detail? | Y | Y | - | Y |
| 9 | Did the review authors use a satisfactory technique for assessing the risk of bias (RoB) in individual studies that were included in the review? | Y | Y | - | Y |
| 10 | Did the review authors report on the sources of funding for the studies included in the review? | N | N | - | N |
| 11 | If meta-analysis was performed, did the review authors use appropriate methods for statistical combination of results? | Y | Y | - | Y |
| 12 | If meta-analysis was performed, did the review authors assess the potential impact of RoB in individual studies on the results of the meta-analysis or other evidence synthesis? | PY | PY | - | PY |
| 13 | Did the review authors account for RoB in primary studies when interpreting/discussing the results of the review? | PY | PY | - | PY |
| 14 | Did the review authors provide a satisfactory explanation for, and discussion of, any heterogeneity observed in the results of the review? | PY | PY | - | PY |
| 15 | If they performed quantitative synthesis did the review authors carry out an adequate investigation of publication bias (small study bias) and discuss its likely impact on the results of the review? | N | N | - | N |
| 16 | Did the review authors report any potential sources of conflict of interest, including any funding they received for conducting the review? | Y | Y | - | Y |

Study2：Werner MT, Bosso JV. Intralymphatic immunotherapy for allergic rhinitis: A systematic review and meta-analysis. Allergy Asthma Proc. 2021 Jul 1;42(4):283-292. doi: 10.2500/aap.2021.42.210028. PMID: 34187620.

| Entry | | A | B | C | D |
| --- | --- | --- | --- | --- | --- |
| 1 | Did the research questions and inclusion criteria for the review include the components of PICO? | Y | Y | - | Y |
| 2 | Did the report of the review contain an explicit statement that the review methods were established prior to the conduct of the review and did the report justify any significantdeviations from the protocol? | N | N | - | N |
| 3 | Did the review authors explain their selection of the study designs for inclusion in the review? | Y | Y | - | Y |
| 4 | Did the review authors use a comprehensive literature search strategy? | Y | Y | - | Y |
| 5 | Did the review authors perform study selection in duplicate? | N | PY | PY | PY |
| 6 | Did the review authors perform data extraction in duplicate? | N | PY | PY | PY |
| 7 | Did the review authors provide a list of excluded studies and justify the exclusions? | PY | Y | Y | Y |
| 8 | Did the review authors describe the included studies in adequate detail? | Y | Y | - | Y |
| 9 | Did the review authors use a satisfactory technique for assessing the risk of bias (RoB) in individual studies that were included in the review? | Y | Y | - | Y |
| 10 | Did the review authors report on the sources of funding for the studies included in the review? | N | N | - | N |
| 11 | If meta-analysis was performed, did the review authors use appropriate methods for statistical combination of results? | Y | Y | - | Y |
| 12 | If meta-analysis was performed, did the review authors assess the potential impact of RoB in individual studies on the results of the meta-analysis or other evidence synthesis? | PY | PY | - | PY |
| 13 | Did the review authors account for RoB in primary studies when interpreting/discussing the results of the review? | Y | PY | Y | Y |
| 14 | Did the review authors provide a satisfactory explanation for, and discussion of, any heterogeneity observed in the results of the review? | Y | Y | - | Y |
| 15 | If they performed quantitative synthesis did the review authors carry out an adequate investigation of publication bias (small study bias) and discuss its likely impact on the results of the review? | PY | Y | Y | Y |
| 16 | Did the review authors report any potential sources of conflict of interest, including any funding they received for conducting the review? | N | Y | Y | Y |

Study3：Hoang MP, Seresirikachorn K, Chitsuthipakorn W, Snidvongs K. Intralymphatic immunotherapy for allergic rhinoconjunctivitis: a systematic review and meta-analysis. Rhinology. 2021 Jun 1;59(3):236-244. doi: 10.4193/Rhin20.572. PMID: 33647073.

| Entry | | A | B | C | D |
| --- | --- | --- | --- | --- | --- |
| 1 | Did the research questions and inclusion criteria for the review include the components of PICO? | Y | Y | - | Y |
| 2 | Did the report of the review contain an explicit statement that the review methods were established prior to the conduct of the review and did the report justify any significantdeviations from the protocol? | Y | Y | - | Y |
| 3 | Did the review authors explain their selection of the study designs for inclusion in the review? | Y | Y | - | Y |
| 4 | Did the review authors use a comprehensive literature search strategy? | Y | Y | - | Y |
| 5 | Did the review authors perform study selection in duplicate? | Y | Y | - | Y |
| 6 | Did the review authors perform data extraction in duplicate? | Y | Y | - | Y |
| 7 | Did the review authors provide a list of excluded studies and justify the exclusions? | PY | PY | - | PY |
| 8 | Did the review authors describe the included studies in adequate detail? | Y | Y | - | Y |
| 9 | Did the review authors use a satisfactory technique for assessing the risk of bias (RoB) in individual studies that were included in the review? | Y | Y | - | Y |
| 10 | Did the review authors report on the sources of funding for the studies included in the review? | N | N | - | N |
| 11 | If meta-analysis was performed, did the review authors use appropriate methods for statistical combination of results? | Y | Y | - | Y |
| 12 | If meta-analysis was performed, did the review authors assess the potential impact of RoB in individual studies on the results of the meta-analysis or other evidence synthesis? | PY | PY | - | PY |
| 13 | Did the review authors account for RoB in primary studies when interpreting/discussing the results of the review? | PY | PY | - | PY |
| 14 | Did the review authors provide a satisfactory explanation for, and discussion of, any heterogeneity observed in the results of the review? | Y | Y | - | Y |
| 15 | If they performed quantitative synthesis did the review authors carry out an adequate investigation of publication bias (small study bias) and discuss its likely impact on the results of the review? | N | N | - | N |
| 16 | Did the review authors report any potential sources of conflict of interest, including any funding they received for conducting the review? | Y | Y | - | Y |

Study 4：Jiang S, Xie S, Tang Q, Zhang H, Xie Z, Zhang J, Jiang W. Evaluation of Intralymphatic Immunotherapy in Allergic Rhinitis Patients: A Systematic Review and Meta-analysis. Mediators Inflamm. 2023 May 8;2023:9377518. doi: 10.1155/2023/9377518. PMID: 37197570; PMCID: PMC10185423.

| Entry | | A | B | C | D |
| --- | --- | --- | --- | --- | --- |
| 1 | Did the research questions and inclusion criteria for the review include the components of PICO? | Y | Y | - | Y |
| 2 | Did the report of the review contain an explicit statement that the review methods were established prior to the conduct of the review and did the report justify any significantdeviations from the protocol? | Y | PY | Y | Y |
| 3 | Did the review authors explain their selection of the study designs for inclusion in the review? | Y | Y | - | Y |
| 4 | Did the review authors use a comprehensive literature search strategy? | Y | Y | - | Y |
| 5 | Did the review authors perform study selection in duplicate? | Y | Y | - | Y |
| 6 | Did the review authors perform data extraction in duplicate? | Y | Y | - | Y |
| 7 | Did the review authors provide a list of excluded studies and justify the exclusions? | PY | PY | - | PY |
| 8 | Did the review authors describe the included studies in adequate detail? | Y | Y | - | Y |
| 9 | Did the review authors use a satisfactory technique for assessing the risk of bias (RoB) in individual studies that were included in the review? | Y | Y | - | Y |
| 10 | Did the review authors report on the sources of funding for the studies included in the review? | N | N | - | N |
| 11 | If meta-analysis was performed, did the review authors use appropriate methods for statistical combination of results? | Y | Y | - | Y |
| 12 | If meta-analysis was performed, did the review authors assess the potential impact of RoB in individual studies on the results of the meta-analysis or other evidence synthesis? | PY | PY | - | PY |
| 13 | Did the review authors account for RoB in primary studies when interpreting/discussing the results of the review? | PY | PY | - | PY |
| 14 | Did the review authors provide a satisfactory explanation for, and discussion of, any heterogeneity observed in the results of the review? | Y | Y | - | Y |
| 15 | If they performed quantitative synthesis did the review authors carry out an adequate investigation of publication bias (small study bias) and discuss its likely impact on the results of the review? | N | PY | PY | PY |
| 16 | Did the review authors report any potential sources of conflict of interest, including any funding they received for conducting the review? | Y | Y | - | Y |

Study 5：Wang W, Wang X, Wang H, Wang X. Evaluation of Safety, Efficacy, and Compliance of Intralymphatic Immunotherapy for Allergic Rhinoconjunctivitis: A Systematic Review and Meta-Analysis. Int Arch Allergy Immunol. 2023;184(8):754-766. doi: 10.1159/000529025. Epub 2023 Apr 27. PMID: 37105134.

| Entry | | A | B | C | D |
| --- | --- | --- | --- | --- | --- |
| 1 | Did the research questions and inclusion criteria for the review include the components of PICO? | Y | Y | - | Y |
| 2 | Did the report of the review contain an explicit statement that the review methods were established prior to the conduct of the review and did the report justify any significantdeviations from the protocol? | PY | PY | - | PY |
| 3 | Did the review authors explain their selection of the study designs for inclusion in the review? | Y | Y | - | Y |
| 4 | Did the review authors use a comprehensive literature search strategy? | Y | Y | - | Y |
| 5 | Did the review authors perform study selection in duplicate? | Y | Y | - | Y |
| 6 | Did the review authors perform data extraction in duplicate? | Y | Y | - | Y |
| 7 | Did the review authors provide a list of excluded studies and justify the exclusions? | N | N | - | N |
| 8 | Did the review authors describe the included studies in adequate detail? | Y | Y | - | Y |
| 9 | Did the review authors use a satisfactory technique for assessing the risk of bias (RoB) in individual studies that were included in the review? | Y | Y | - | Y |
| 10 | Did the review authors report on the sources of funding for the studies included in the review? | N | N | - | N |
| 11 | If meta-analysis was performed, did the review authors use appropriate methods for statistical combination of results? | Y | Y | - | Y |
| 12 | If meta-analysis was performed, did the review authors assess the potential impact of RoB in individual studies on the results of the meta-analysis or other evidence synthesis? | PY | PY | - | PY |
| 13 | Did the review authors account for RoB in primary studies when interpreting/discussing the results of the review? | PY | PY | - | PY |
| 14 | Did the review authors provide a satisfactory explanation for, and discussion of, any heterogeneity observed in the results of the review? | Y | PY | PY | PY |
| 15 | If they performed quantitative synthesis did the review authors carry out an adequate investigation of publication bias (small study bias) and discuss its likely impact on the results of the review? | N | N | - | N |
| 16 | Did the review authors report any potential sources of conflict of interest, including any funding they received for conducting the review? | Y | Y | - | Y |

Study 6：Liu L, Liang Y, Yan L, Li Z. Is intralymphatic immunotherapy effective and safe for allergic rhinitis?: A meta-analysis. Medicine (Baltimore). 2024 Nov 15;103(46):e40589. doi: 10.1097/MD.0000000000040589. PMID: 39560522; PMCID: PMC11576000.

| Entry | | A | B | C | D |
| --- | --- | --- | --- | --- | --- |
| 1 | Did the research questions and inclusion criteria for the review include the components of PICO? | Y | Y | - | Y |
| 2 | Did the report of the review contain an explicit statement that the review methods were established prior to the conduct of the review and did the report justify any significantdeviations from the protocol? | Y | PY | Y | Y |
| 3 | Did the review authors explain their selection of the study designs for inclusion in the review? | Y | Y | - | Y |
| 4 | Did the review authors use a comprehensive literature search strategy? | Y | Y | - | Y |
| 5 | Did the review authors perform study selection in duplicate? | Y | Y | - | Y |
| 6 | Did the review authors perform data extraction in duplicate? | Y | Y | - | Y |
| 7 | Did the review authors provide a list of excluded studies and justify the exclusions? | N | N | - | N |
| 8 | Did the review authors describe the included studies in adequate detail? | Y | Y | - | Y |
| 9 | Did the review authors use a satisfactory technique for assessing the risk of bias (RoB) in individual studies that were included in the review? | Y | Y | - | Y |
| 10 | Did the review authors report on the sources of funding for the studies included in the review? | N | N | - | N |
| 11 | If meta-analysis was performed, did the review authors use appropriate methods for statistical combination of results? | Y | Y | - | Y |
| 12 | If meta-analysis was performed, did the review authors assess the potential impact of RoB in individual studies on the results of the meta-analysis or other evidence synthesis? | PY | PY | - | PY |
| 13 | Did the review authors account for RoB in primary studies when interpreting/discussing the results of the review? | PY | PY | - | PY |
| 14 | Did the review authors provide a satisfactory explanation for, and discussion of, any heterogeneity observed in the results of the review? | Y | Y | - | Y |
| 15 | If they performed quantitative synthesis did the review authors carry out an adequate investigation of publication bias (small study bias) and discuss its likely impact on the results of the review? | Y | Y | - | Y |
| 16 | Did the review authors report any potential sources of conflict of interest, including any funding they received for conducting the review? | N | Y | Y | Y |

Study 7：曾婉婷,李艮平,蒋建国,等.淋巴结内特异性免疫治疗变应性鼻炎疗效和安全性的Meta分析[J].中国耳鼻咽喉颅底外科杂志,2024,30(02):9-18.

| Entry | | A | B | C | D |
| --- | --- | --- | --- | --- | --- |
| 1 | Did the research questions and inclusion criteria for the review include the components of PICO? | Y | Y | - | Y |
| 2 | Did the report of the review contain an explicit statement that the review methods were established prior to the conduct of the review and did the report justify any significantdeviations from the protocol? | PY | N | PY | PY |
| 3 | Did the review authors explain their selection of the study designs for inclusion in the review? | Y | Y | - | Y |
| 4 | Did the review authors use a comprehensive literature search strategy? | Y | Y | - | Y |
| 5 | Did the review authors perform study selection in duplicate? | Y | Y | - | Y |
| 6 | Did the review authors perform data extraction in duplicate? | Y | Y | - | Y |
| 7 | Did the review authors provide a list of excluded studies and justify the exclusions? | PY | Y | PY | PY |
| 8 | Did the review authors describe the included studies in adequate detail? | Y | Y | - | Y |
| 9 | Did the review authors use a satisfactory technique for assessing the risk of bias (RoB) in individual studies that were included in the review? | Y | Y | - | Y |
| 10 | Did the review authors report on the sources of funding for the studies included in the review? | N | N | - | N |
| 11 | If meta-analysis was performed, did the review authors use appropriate methods for statistical combination of results? | Y | Y | - | Y |
| 12 | If meta-analysis was performed, did the review authors assess the potential impact of RoB in individual studies on the results of the meta-analysis or other evidence synthesis? | PY | PY | - | PY |
| 13 | Did the review authors account for RoB in primary studies when interpreting/discussing the results of the review? | PY | PY | - | PY |
| 14 | Did the review authors provide a satisfactory explanation for, and discussion of, any heterogeneity observed in the results of the review? | Y | Y | - | Y |
| 15 | If they performed quantitative synthesis did the review authors carry out an adequate investigation of publication bias (small study bias) and discuss its likely impact on the results of the review? | N | N | - | N |
| 16 | Did the review authors report any potential sources of conflict of interest, including any funding they received for conducting the review? | N | N | - | N |

4.PRISMA 2020

Y: Yes; N: No; PY: Partial Yes.

A:The conclusions of researcher Ph.D. Zhuang Wang.

B:The conclusions of researcher Ph.D. Xiaofei Xie.

C:In case of a difference of opinions, it shall be adjudicated by Professor Zhikai Qiu.

D:Conclusive conclusion.

Study 1：Aini NR, Mohd Noor N, Md Daud MK, Wise SK, Abdullah B. Efficacy and safety of intralymphatic immunotherapy in allergic rhinitis: A systematic review and meta-analysis. Clin Transl Allergy. 2021 Aug 17;11(6):e12055. doi: 10.1002/clt2.12055. PMID: 34429875; PMCID: PMC8369948.

Table 1：

| Section and topic | Item # | Checklist item | A | B | C | D |
| --- | --- | --- | --- | --- | --- | --- |
| **Title** | | | | | | |
| Title | 1 | Identify the report as a systematic review. | Y | Y | - | Y |
| **Abstract** | | | | | | |
| Abstract | 2 | See the PRISMA 2020 for Abstracts checklist (table 2). | PY | PY | - | PY |
| **Introduction** | | | | | | |
| Rationale | 3 | Describe the rationale for the review in the context of existing knowledge | Y | Y | - | Y |
| Objectives | 4 | Provide an explicit statement of the objective(s) or question(s) the review addresses. | Y | Y | - | Y |
| **Methods** | | | | | | |
| Eligibility criteria | 5 | Specify the inclusion and exclusion criteria for the review and how studies were grouped for the syntheses. | Y | Y | - | Y |
| Information sources | 6 | Specify all databases, registers, websites, organisations, reference lists and other sources searched or consulted to identify studies. Specify the date when each source was last searched or consulted. | Y | Y | - | Y |
| Search strategy | 7 | Present the full search strategies for all databases, registers and websites, including any filters and limits used | Y | Y | - | Y |
| Selection process | 8 | Specify the methods used to decide whether a study met the inclusion criteria of the review, including how many reviewers screened each record and each report retrieved, whether they worked independently, and if applicable, details of automation tools  used in the process. | Y | Y | - | Y |
| Data collection  process | 9 | Specify the methods used to collect data from reports, including how many reviewers collected data from each report, whether they worked independently, any processes for obtaining or confirming data from study investigators, and if applicable, details of automation tools used in the process. | Y | Y | - | Y |
| Data items | 10a | List and define all outcomes for which data were sought. Specify whether all results that were compatible with each outcome domain in each study were sought (e.g. for all measures, time points, analyses), and if not, the methods used to decide which  results to collect. | Y | Y | - | Y |
|  | 10b | List and define all other variables for which data were sought (e.g. participant and intervention characteristics, funding sources). Describe any assumptions made about any missing or unclear information. | Y | Y | - | Y |
| Study risk of bias  assessment | 11 | Specify the methods used to assess risk of bias in the included studies, including details of the tool(s) used, how many reviewers assessed each study and whether they worked independently, and if applicable, details of automation tools used in the process. | Y | Y | - | Y |
| Effect measures | 12 | Specify for each outcome the effect measure(s) (e.g. risk ratio, mean difference) used in the synthesis or presentation of results. | Y | Y | - | Y |
| Synthesis methods | 13a | Describe the processes used to decide which studies were eligible for each synthesis (e.g. tabulating the study intervention characteristics and comparing against the planned groups for each synthesis (item #5)). | Y | Y | - | Y |
|  | 13b | Describe any methods required to prepare the data for presentation or synthesis, such as handling of missing summary statistics, or data conversions. | Y | Y | - | Y |
|  | 13c | Describe any methods used to tabulate or visually display results of individual studies and syntheses. | Y | Y | - | Y |
|  | 13d | Describe any methods used to synthesise results and provide a rationale for the choice(s). If meta-analysis was performed, describe the model(s), method(s) to identify the presence and extent of statistical heterogeneity, and software package(s) used. | Y | Y | - | Y |
|  | 13e | Describe any methods used to explore possible causes of heterogeneity among study results (e.g. subgroup analysis, meta regression). | Y | Y | - | Y |
|  | 13f | Describe any sensitivity analyses conducted to assess robustness of the synthesised results. | PY | N | N | N |
| Reporting bias  assessment | 14 | Describe any methods used to assess risk of bias due to missing results in a synthesis (arising from reporting biases). | Y | Y | - | Y |
| Certainty assessment | 15 | Describe any methods used to assess certainty (or confidence) in the body of evidence for an outcome. | Y | Y | - | Y |
| **Results** | | | | | | |
| Study selection | 16a | Describe the results of the search and selection process, from the number of records identified in the search to the number of studies included in the review, ideally using a flow diagram (see fig 1). | Y | Y | - | Y |
|  | 16b | Cite studies that might appear to meet the inclusion criteria, but which were excluded, and explain why they were excluded. | Y | Y | - | Y |
| Study characteristics | 17 | Cite each included study and present its characteristics. | Y | Y | - | Y |
| Risk of bias in studies | 18 | Present assessments of risk of bias for each included study. | Y | Y | - | Y |
| Results of individual  studies | 19 | For all outcomes, present, for each study: (a) summary statistics for each group (where appropriate) and (b) an effect estimate and its precision (e.g. confidence/credible interval), ideally using structured tables or plots. | Y | Y | - | Y |
| Results of syntheses | 20a | For each synthesis, briefly summarise the characteristics and risk of bias among contributing studies. | Y | Y | - | Y |
|  | 20b | Present results of all statistical syntheses conducted. If meta-analysis was done, present for each the summary estimate and its precision (e.g. confidence/credible interval) and measures of statistical heterogeneity. If comparing groups, describe the direction of the effect. | Y | Y | - | Y |
|  | 20c | Present results of all investigations of possible causes of heterogeneity among study results. | Y | Y | - | Y |
|  | 20d | Present results of all sensitivity analyses conducted to assess the robustness of the synthesised results. | PY | PY | - | PY |
| Reporting biases | 21 | Present assessments of risk of bias due to missing results (arising from reporting biases) for each synthesis assessed. | Y | Y | - | Y |
| Certainty of evidence | 22 | Present assessments of certainty (or confidence) in the body of evidence for each outcome assessed. | Y | Y | - | Y |
| **Discussion** | | | | | | |
| Discussion | 23a | Provide a general interpretation of the results in the context of other evidence. | Y | Y | - | Y |
|  | 23b | Discuss any limitations of the evidence included in the review. | Y | Y | - | Y |
|  | 23c | Discuss any limitations of the review processes used. | Y | Y | - | Y |
|  | 23d | Discuss implications of the results for practice, policy, and future research. | Y | Y | - | Y |
| **Other information** | | | | | | |
| Registration and  protocol | 24a | Provide registration information for the review, including register name and registration number, or state that the review was not registered. | Y | Y | - | Y |
|  | 24b | Indicate where the review protocol can be accessed, or state that a protocol was not prepared. | Y | Y | - | Y |
|  | 24c | Describe and explain any amendments to information provided at registration or in the protocol. | PY | N | PY | PY |
| Support | 25 | Describe sources of financial or non-financial support for the review, and the role of the funders or sponsors in the review. | Y | Y | - | Y |
| Competing interests | 26 | Declare any competing interests of review authors. | Y | Y | - | Y |
| Availability of data,  code, and other  materials | 27 | Report which of the following are publicly available and where they can be found: template data collection forms; data extracted from included studies; data used for all analyses; analytic code; any other materials used in the review. | PY | Y | N | N |

Table 2：

| Section and topic | Item # | Checklist item | A | B | C | D |
| --- | --- | --- | --- | --- | --- | --- |
| **Title** | | | | | | |
| Title | 1 | Identify the report as a systematic review. | Y | Y | - | Y |
| **Background** | | | | | | |
| Objectives | 2 | Provide an explicit statement of the main objective(s) or question(s) the review addresses. | Y | Y | - | Y |
| **Methods** | | | | | | |
| Eligibility criteria | 3 | Specify the inclusion and exclusion criteria for the review | Y | Y | - | Y |
| Information sources | 4 | Specify the information sources (e.g. databases, registers) used to identify studies and the date when each was last searched. | Y | Y | - | Y |
| Risk of bias | 5 | Specify the methods used to assess risk of bias in the included studies. | Y | Y | - | Y |
| Synthesis of results | 6 | Specify the methods used to present and synthesise results. | Y | Y | - | Y |
| **Results** | | | | | | |
| Included studies | 7 | Give the total number of included studies and participants and summarise relevant characteristics of studies. | Y | Y | - | Y |
| Synthesis of results | 8 | Present results for main outcomes, preferably indicating the number of included studies and participants for each. If meta-analysis was done, report the summary estimate and confidence/credible interval. If comparing groups, indicate the direction of the effect (i.e. which group is favoured). | Y | Y | - | Y |
| **Discussion** | | | | | | |
| Limitations of evidence | 9 | Provide a brief summary of the limitations of the evidence included in the review (e.g. study risk of bias, inconsistency and imprecision). | Y | Y | - | Y |
| Interpretation | 10 | Provide a general interpretation of the results and important implications. | Y | Y | - | Y |
| **Other** | | | | | | |
| Funding | 11 | Specify the primary source of funding for the review. | PY | N | N | N |
| Registration | 12 | Provide the register name and registration number. | Y | Y | - | Y |

Study 2：Werner MT, Bosso JV. Intralymphatic immunotherapy for allergic rhinitis: A systematic review and meta-analysis. Allergy Asthma Proc. 2021 Jul 1;42(4):283-292. doi: 10.2500/aap.2021.42.210028. PMID: 34187620.

Table 1：

| Section and topic | Item # | Checklist item | A | B | C | D |
| --- | --- | --- | --- | --- | --- | --- |
| **Title** | | | | | | |
| Title | 1 | Identify the report as a systematic review. | Y | Y | - | Y |
| **Abstract** | | | | | | |
| Abstract | 2 | See the PRISMA 2020 for Abstracts checklist (table 2). | PY | PY | - | PY |
| **Introduction** | | | | | | |
| Rationale | 3 | Describe the rationale for the review in the context of existing knowledge | Y | Y | - | Y |
| Objectives | 4 | Provide an explicit statement of the objective(s) or question(s) the review addresses. | Y | Y | - | Y |
| **Methods** | | | | | | |
| Eligibility criteria | 5 | Specify the inclusion and exclusion criteria for the review and how studies were grouped for the syntheses. | Y | Y | - | Y |
| Information sources | 6 | Specify all databases, registers, websites, organisations, reference lists and other sources searched or consulted to identify studies. Specify the date when each source was last searched or consulted. | Y | Y | - | Y |
| Search strategy | 7 | Present the full search strategies for all databases, registers and websites, including any filters and limits used | Y | Y | - | Y |
| Selection process | 8 | Specify the methods used to decide whether a study met the inclusion criteria of the review, including how many reviewers screened each record and each report retrieved, whether they worked independently, and if applicable, details of automation tools  used in the process. | Y | Y | - | Y |
| Data collection  process | 9 | Specify the methods used to collect data from reports, including how many reviewers collected data from each report, whether they worked independently, any processes for obtaining or confirming data from study investigators, and if applicable, details of automation tools used in the process. | Y | Y | - | Y |
| Data items | 10a | List and define all outcomes for which data were sought. Specify whether all results that were compatible with each outcome domain in each study were sought (e.g. for all measures, time points, analyses), and if not, the methods used to decide which  results to collect. | Y | Y | - | Y |
|  | 10b | List and define all other variables for which data were sought (e.g. participant and intervention characteristics, funding sources). Describe any assumptions made about any missing or unclear information. | Y | Y | - | Y |
| Study risk of bias  assessment | 11 | Specify the methods used to assess risk of bias in the included studies, including details of the tool(s) used, how many reviewers assessed each study and whether they worked independently, and if applicable, details of automation tools used in the process. | Y | Y | - | Y |
| Effect measures | 12 | Specify for each outcome the effect measure(s) (e.g. risk ratio, mean difference) used in the synthesis or presentation of results. | Y | Y | - | Y |
| Synthesis methods | 13a | Describe the processes used to decide which studies were eligible for each synthesis (e.g. tabulating the study intervention characteristics and comparing against the planned groups for each synthesis (item #5)). | PY | Y | Y | Y |
|  | 13b | Describe any methods required to prepare the data for presentation or synthesis, such as handling of missing summary statistics, or data conversions. | PY | Y | Y | Y |
|  | 13c | Describe any methods used to tabulate or visually display results of individual studies and syntheses. | PY | Y | Y | Y |
|  | 13d | Describe any methods used to synthesise results and provide a rationale for the choice(s). If meta-analysis was performed, describe the model(s), method(s) to identify the presence and extent of statistical heterogeneity, and software package(s) used. | PY | PY | - | PY |
|  | 13e | Describe any methods used to explore possible causes of heterogeneity among study results (e.g. subgroup analysis, meta regression). | PY | PY | - | PY |
|  | 13f | Describe any sensitivity analyses conducted to assess robustness of the synthesised results. | PY | PY | - | PY |
| Reporting bias  assessment | 14 | Describe any methods used to assess risk of bias due to missing results in a synthesis (arising from reporting biases). | PY | Y | PY | PY |
| Certainty assessment | 15 | Describe any methods used to assess certainty (or confidence) in the body of evidence for an outcome. | PY | Y | N | N |
| **Results** | | | | | | |
| Study selection | 16a | Describe the results of the search and selection process, from the number of records identified in the search to the number of studies included in the review, ideally using a flow diagram (see fig 1). | Y | Y | - | Y |
|  | 16b | Cite studies that might appear to meet the inclusion criteria, but which were excluded, and explain why they were excluded. | Y | N | Y | Y |
| Study characteristics | 17 | Cite each included study and present its characteristics. | Y | Y | - | Y |
| Risk of bias in studies | 18 | Present assessments of risk of bias for each included study. | Y | Y | - | Y |
| Results of individual  studies | 19 | For all outcomes, present, for each study: (a) summary statistics for each group (where appropriate) and (b) an effect estimate and its precision (e.g. confidence/credible interval), ideally using structured tables or plots. | Y | Y | - | Y |
| Results of syntheses | 20a | For each synthesis, briefly summarise the characteristics and risk of bias among contributing studies. | Y | Y | - | Y |
|  | 20b | Present results of all statistical syntheses conducted. If meta-analysis was done, present for each the summary estimate and its precision (e.g. confidence/credible interval) and measures of statistical heterogeneity. If comparing groups, describe the direction of the effect. | Y | Y | - | Y |
|  | 20c | Present results of all investigations of possible causes of heterogeneity among study results. | Y | Y | - | Y |
|  | 20d | Present results of all sensitivity analyses conducted to assess the robustness of the synthesised results. | Y | Y | - | Y |
| Reporting biases | 21 | Present assessments of risk of bias due to missing results (arising from reporting biases) for each synthesis assessed. | Y | Y | - | Y |
| Certainty of evidence | 22 | Present assessments of certainty (or confidence) in the body of evidence for each outcome assessed. | Y | Y | - | Y |
| **Discussion** | | | | | | |
| Discussion | 23a | Provide a general interpretation of the results in the context of other evidence. | Y | Y | - | Y |
|  | 23b | Discuss any limitations of the evidence included in the review. | Y | Y | - | Y |
|  | 23c | Discuss any limitations of the review processes used. | Y | Y | - | Y |
|  | 23d | Discuss implications of the results for practice, policy, and future research. | Y | Y | - | Y |
| **Other information** | | | | | | |
| Registration and  protocol | 24a | Provide registration information for the review, including register name and registration number, or state that the review was not registered. | N | N | - | N |
|  | 24b | Indicate where the review protocol can be accessed, or state that a protocol was not prepared. | N | N | - | N |
|  | 24c | Describe and explain any amendments to information provided at registration or in the protocol. | N | N | - | N |
| Support | 25 | Describe sources of financial or non-financial support for the review, and the role of the funders or sponsors in the review. | Y | Y | - | Y |
| Competing interests | 26 | Declare any competing interests of review authors. | Y | Y | - | Y |
| Availability of data,  code, and other  materials | 27 | Report which of the following are publicly available and where they can be found: template data collection forms; data extracted from included studies; data used for all analyses; analytic code; any other materials used in the review. | N | Y | PY | PY |

Table 2：

| Section and topic | Item # | Checklist item | A | B | C | D |
| --- | --- | --- | --- | --- | --- | --- |
| **Title** | | | | | | |
| Title | 1 | Identify the report as a systematic review. | Y | Y | - | Y |
| **Background** | | | | | | |
| Objectives | 2 | Provide an explicit statement of the main objective(s) or question(s) the review addresses. | Y | Y | - | Y |
| **Methods** | | | | | | |
| Eligibility criteria | 3 | Specify the inclusion and exclusion criteria for the review | Y | Y | - | Y |
| Information sources | 4 | Specify the information sources (e.g. databases, registers) used to identify studies and the date when each was last searched. | Y | Y | - | Y |
| Risk of bias | 5 | Specify the methods used to assess risk of bias in the included studies. | Y | Y | - | Y |
| Synthesis of results | 6 | Specify the methods used to present and synthesise results. | Y | Y | - | Y |
| **Results** | | | | | | |
| Included studies | 7 | Give the total number of included studies and participants and summarise relevant characteristics of studies. | Y | Y | - | Y |
| Synthesis of results | 8 | Present results for main outcomes, preferably indicating the number of included studies and participants for each. If meta-analysis was done, report the summary estimate and confidence/credible interval. If comparing groups, indicate the direction of the effect (i.e. which group is favoured). | Y | Y | - | Y |
| **Discussion** | | | | | | |
| Limitations of evidence | 9 | Provide a brief summary of the limitations of the evidence included in the review (e.g. study risk of bias, inconsistency and imprecision). | Y | Y | - | Y |
| Interpretation | 10 | Provide a general interpretation of the results and important implications. | Y | Y | - | Y |
| **Other** | | | | | | |
| Funding | 11 | Specify the primary source of funding for the review. | N | N | - | N |
| Registration | 12 | Provide the register name and registration number. | N | N | - | N |

Study 3：Hoang MP, Seresirikachorn K, Chitsuthipakorn W, Snidvongs K. Intralymphatic immunotherapy for allergic rhinoconjunctivitis: a systematic review and meta-analysis. Rhinology. 2021 Jun 1;59(3):236-244. doi: 10.4193/Rhin20.572. PMID: 33647073.

Table 1：

| Section and topic | Item # | Checklist item | A | B | C | D |
| --- | --- | --- | --- | --- | --- | --- |
| **Title** | | | | | | |
| Title | 1 | Identify the report as a systematic review. | Y | Y | - | Y |
| **Abstract** | | | | | | |
| Abstract | 2 | See the PRISMA 2020 for Abstracts checklist (table 2). | PY | PY | - | PY |
| **Introduction** | | | | | | |
| Rationale | 3 | Describe the rationale for the review in the context of existing knowledge | Y | Y | - | Y |
| Objectives | 4 | Provide an explicit statement of the objective(s) or question(s) the review addresses. | Y | Y | - | Y |
| **Methods** | | | | | | |
| Eligibility criteria | 5 | Specify the inclusion and exclusion criteria for the review and how studies were grouped for the syntheses. | Y | Y | - | Y |
| Information sources | 6 | Specify all databases, registers, websites, organisations, reference lists and other sources searched or consulted to identify studies. Specify the date when each source was last searched or consulted. | Y | Y | - | Y |
| Search strategy | 7 | Present the full search strategies for all databases, registers and websites, including any filters and limits used | Y | Y | - | Y |
| Selection process | 8 | Specify the methods used to decide whether a study met the inclusion criteria of the review, including how many reviewers screened each record and each report retrieved, whether they worked independently, and if applicable, details of automation tools  used in the process. | Y | Y | - | Y |
| Data collection  process | 9 | Specify the methods used to collect data from reports, including how many reviewers collected data from each report, whether they worked independently, any processes for obtaining or confirming data from study investigators, and if applicable, details of automation tools used in the process. | Y | Y | - | Y |
| Data items | 10a | List and define all outcomes for which data were sought. Specify whether all results that were compatible with each outcome domain in each study were sought (e.g. for all measures, time points, analyses), and if not, the methods used to decide which  results to collect. | PY | Y | Y | Y |
|  | 10b | List and define all other variables for which data were sought (e.g. participant and intervention characteristics, funding sources). Describe any assumptions made about any missing or unclear information. | PY | Y | Y | Y |
| Study risk of bias  assessment | 11 | Specify the methods used to assess risk of bias in the included studies, including details of the tool(s) used, how many reviewers assessed each study and whether they worked independently, and if applicable, details of automation tools used in the process. | Y | PY | Y | Y |
| Effect measures | 12 | Specify for each outcome the effect measure(s) (e.g. risk ratio, mean difference) used in the synthesis or presentation of results. | Y | Y | - | Y |
| Synthesis methods | 13a | Describe the processes used to decide which studies were eligible for each synthesis (e.g. tabulating the study intervention characteristics and comparing against the planned groups for each synthesis (item #5)). | Y | Y | - | Y |
|  | 13b | Describe any methods required to prepare the data for presentation or synthesis, such as handling of missing summary statistics, or data conversions. | PY | Y | N | N |
|  | 13c | Describe any methods used to tabulate or visually display results of individual studies and syntheses. | Y | Y | - | Y |
|  | 13d | Describe any methods used to synthesise results and provide a rationale for the choice(s). If meta-analysis was performed, describe the model(s), method(s) to identify the presence and extent of statistical heterogeneity, and software package(s) used. | Y | Y | - | Y |
|  | 13e | Describe any methods used to explore possible causes of heterogeneity among study results (e.g. subgroup analysis, meta regression). | PY | Y | PY | PY |
|  | 13f | Describe any sensitivity analyses conducted to assess robustness of the synthesised results. | PY | PY | - | PY |
| Reporting bias  assessment | 14 | Describe any methods used to assess risk of bias due to missing results in a synthesis (arising from reporting biases). | N | Y | N | N |
| Certainty assessment | 15 | Describe any methods used to assess certainty (or confidence) in the body of evidence for an outcome. | N | PY | N | N |
| **Results** | | | | | | |
| Study selection | 16a | Describe the results of the search and selection process, from the number of records identified in the search to the number of studies included in the review, ideally using a flow diagram (see fig 1). | Y | Y | - | Y |
|  | 16b | Cite studies that might appear to meet the inclusion criteria, but which were excluded, and explain why they were excluded. | Y | Y | - | Y |
| Study characteristics | 17 | Cite each included study and present its characteristics. | Y | Y | - | Y |
| Risk of bias in studies | 18 | Present assessments of risk of bias for each included study. | Y | Y | - | Y |
| Results of individual  studies | 19 | For all outcomes, present, for each study: (a) summary statistics for each group (where appropriate) and (b) an effect estimate and its precision (e.g. confidence/credible interval), ideally using structured tables or plots. | Y | Y | - | Y |
| Results of syntheses | 20a | For each synthesis, briefly summarise the characteristics and risk of bias among contributing studies. | Y | Y | - | Y |
|  | 20b | Present results of all statistical syntheses conducted. If meta-analysis was done, present for each the summary estimate and its precision (e.g. confidence/credible interval) and measures of statistical heterogeneity. If comparing groups, describe the direction of the effect. | Y | Y | - | Y |
|  | 20c | Present results of all investigations of possible causes of heterogeneity among study results. | Y | Y | - | Y |
|  | 20d | Present results of all sensitivity analyses conducted to assess the robustness of the synthesised results. | Y | PY | N | N |
| Reporting biases | 21 | Present assessments of risk of bias due to missing results (arising from reporting biases) for each synthesis assessed. | Y | Y | - | Y |
| Certainty of evidence | 22 | Present assessments of certainty (or confidence) in the body of evidence for each outcome assessed. | N | PY | N | N |
| **Discussion** | | | | | | |
| Discussion | 23a | Provide a general interpretation of the results in the context of other evidence. | Y | Y | - | Y |
|  | 23b | Discuss any limitations of the evidence included in the review. | Y | Y | - | Y |
|  | 23c | Discuss any limitations of the review processes used. | PY | PY | - | PY |
|  | 23d | Discuss implications of the results for practice, policy, and future research. | Y | Y | - | Y |
| **Other information** | | | | | | |
| Registration and  protocol | 24a | Provide registration information for the review, including register name and registration number, or state that the review was not registered. | Y | Y | - | Y |
|  | 24b | Indicate where the review protocol can be accessed, or state that a protocol was not prepared. | PY | Y | Y | Y |
|  | 24c | Describe and explain any amendments to information provided at registration or in the protocol. | N | N | - | N |
| Support | 25 | Describe sources of financial or non-financial support for the review, and the role of the funders or sponsors in the review. | N | Y | Y | Y |
| Competing interests | 26 | Declare any competing interests of review authors. | N | Y | Y | Y |
| Availability of data,  code, and other  materials | 27 | Report which of the following are publicly available and where they can be found: template data collection forms; data extracted from included studies; data used for all analyses; analytic code; any other materials used in the review. | Y | N | N | N |

Table 2：

| Section and topic | Item # | Checklist item | A | B | C | D |
| --- | --- | --- | --- | --- | --- | --- |
| **Title** | | | | | | |
| Title | 1 | Identify the report as a systematic review. | Y | Y | - | Y |
| **Background** | | | | | | |
| Objectives | 2 | Provide an explicit statement of the main objective(s) or question(s) the review addresses. | Y | Y | - | Y |
| **Methods** | | | | | | |
| Eligibility criteria | 3 | Specify the inclusion and exclusion criteria for the review | Y | Y | - | Y |
| Information sources | 4 | Specify the information sources (e.g. databases, registers) used to identify studies and the date when each was last searched. | Y | Y | - | Y |
| Risk of bias | 5 | Specify the methods used to assess risk of bias in the included studies. | Y | Y | - | Y |
| Synthesis of results | 6 | Specify the methods used to present and synthesise results. | Y | Y | - | Y |
| **Results** | | | | | | |
| Included studies | 7 | Give the total number of included studies and participants and summarise relevant characteristics of studies. | Y | Y | - | Y |
| Synthesis of results | 8 | Present results for main outcomes, preferably indicating the number of included studies and participants for each. If meta-analysis was done, report the summary estimate and confidence/credible interval. If comparing groups, indicate the direction of the effect (i.e. which group is favoured). | Y | Y | - | Y |
| **Discussion** | | | | | | |
| Limitations of evidence | 9 | Provide a brief summary of the limitations of the evidence included in the review (e.g. study risk of bias, inconsistency and imprecision). | Y | Y | - | Y |
| Interpretation | 10 | Provide a general interpretation of the results and important implications. | Y | Y | - | Y |
| **Other** | | | | | | |
| Funding | 11 | Specify the primary source of funding for the review. | N | N | - | N |
| Registration | 12 | Provide the register name and registration number. | N | N | - | N |

Study 4：Jiang S, Xie S, Tang Q, Zhang H, Xie Z, Zhang J, Jiang W. Evaluation of Intralymphatic Immunotherapy in Allergic Rhinitis Patients: A Systematic Review and Meta-analysis. Mediators Inflamm. 2023 May 8;2023:9377518. doi: 10.1155/2023/9377518. PMID: 37197570; PMCID: PMC10185423.

Table 1：

| Section and topic | Item # | Checklist item | A | B | C | D |
| --- | --- | --- | --- | --- | --- | --- |
| **Title** | | | | | | |
| Title | 1 | Identify the report as a systematic review. | Y | Y | - | Y |
| **Abstract** | | | | | | |
| Abstract | 2 | See the PRISMA 2020 for Abstracts checklist (table 2). | PY | PY | - | PY |
| **Introduction** | | | | | | |
| Rationale | 3 | Describe the rationale for the review in the context of existing knowledge | Y | Y | - | Y |
| Objectives | 4 | Provide an explicit statement of the objective(s) or question(s) the review addresses. | Y | Y | - | Y |
| **Methods** | | | | | | |
| Eligibility criteria | 5 | Specify the inclusion and exclusion criteria for the review and how studies were grouped for the syntheses. | Y | Y | - | Y |
| Information sources | 6 | Specify all databases, registers, websites, organisations, reference lists and other sources searched or consulted to identify studies. Specify the date when each source was last searched or consulted. | Y | Y | - | Y |
| Search strategy | 7 | Present the full search strategies for all databases, registers and websites, including any filters and limits used | Y | PY | PY | PY |
| Selection process | 8 | Specify the methods used to decide whether a study met the inclusion criteria of the review, including how many reviewers screened each record and each report retrieved, whether they worked independently, and if applicable, details of automation tools  used in the process. | Y | Y | - | Y |
| Data collection  process | 9 | Specify the methods used to collect data from reports, including how many reviewers collected data from each report, whether they worked independently, any processes for obtaining or confirming data from study investigators, and if applicable, details of automation tools used in the process. | Y | Y | - | Y |
| Data items | 10a | List and define all outcomes for which data were sought. Specify whether all results that were compatible with each outcome domain in each study were sought (e.g. for all measures, time points, analyses), and if not, the methods used to decide which  results to collect. | Y | Y | - | Y |
|  | 10b | List and define all other variables for which data were sought (e.g. participant and intervention characteristics, funding sources). Describe any assumptions made about any missing or unclear information. | Y | Y | - | Y |
| Study risk of bias  assessment | 11 | Specify the methods used to assess risk of bias in the included studies, including details of the tool(s) used, how many reviewers assessed each study and whether they worked independently, and if applicable, details of automation tools used in the process. | Y | Y | - | Y |
| Effect measures | 12 | Specify for each outcome the effect measure(s) (e.g. risk ratio, mean difference) used in the synthesis or presentation of results. | Y | Y | - | Y |
| Synthesis methods | 13a | Describe the processes used to decide which studies were eligible for each synthesis (e.g. tabulating the study intervention characteristics and comparing against the planned groups for each synthesis (item #5)). | Y | Y | - | Y |
|  | 13b | Describe any methods required to prepare the data for presentation or synthesis, such as handling of missing summary statistics, or data conversions. | Y | Y | - | Y |
|  | 13c | Describe any methods used to tabulate or visually display results of individual studies and syntheses. | Y | Y | - | Y |
|  | 13d | Describe any methods used to synthesise results and provide a rationale for the choice(s). If meta-analysis was performed, describe the model(s), method(s) to identify the presence and extent of statistical heterogeneity, and software package(s) used. | Y | Y | - | Y |
|  | 13e | Describe any methods used to explore possible causes of heterogeneity among study results (e.g. subgroup analysis, meta regression). | Y | Y | - | Y |
|  | 13f | Describe any sensitivity analyses conducted to assess robustness of the synthesised results. | Y | Y | - | Y |
| Reporting bias  assessment | 14 | Describe any methods used to assess risk of bias due to missing results in a synthesis (arising from reporting biases). | Y | Y | - | Y |
| Certainty assessment | 15 | Describe any methods used to assess certainty (or confidence) in the body of evidence for an outcome. | Y | Y | - | Y |
| **Results** | | | | | | |
| Study selection | 16a | Describe the results of the search and selection process, from the number of records identified in the search to the number of studies included in the review, ideally using a flow diagram (see fig 1). | Y | Y | - | Y |
|  | 16b | Cite studies that might appear to meet the inclusion criteria, but which were excluded, and explain why they were excluded. | Y | Y | - | Y |
| Study characteristics | 17 | Cite each included study and present its characteristics. | Y | Y | - | Y |
| Risk of bias in studies | 18 | Present assessments of risk of bias for each included study. | Y | Y | - | Y |
| Results of individual  studies | 19 | For all outcomes, present, for each study: (a) summary statistics for each group (where appropriate) and (b) an effect estimate and its precision (e.g. confidence/credible interval), ideally using structured tables or plots. | Y | Y | - | Y |
| Results of syntheses | 20a | For each synthesis, briefly summarise the characteristics and risk of bias among contributing studies. | Y | Y | - | Y |
|  | 20b | Present results of all statistical syntheses conducted. If meta-analysis was done, present for each the summary estimate and its precision (e.g. confidence/credible interval) and measures of statistical heterogeneity. If comparing groups, describe the direction of the effect. | Y | Y | - | Y |
|  | 20c | Present results of all investigations of possible causes of heterogeneity among study results. | Y | Y | - | Y |
|  | 20d | Present results of all sensitivity analyses conducted to assess the robustness of the synthesised results. | Y | Y | - | Y |
| Reporting biases | 21 | Present assessments of risk of bias due to missing results (arising from reporting biases) for each synthesis assessed. | Y | Y | - | Y |
| Certainty of evidence | 22 | Present assessments of certainty (or confidence) in the body of evidence for each outcome assessed. | Y | Y | - | Y |
| **Discussion** | | | | | | |
| Discussion | 23a | Provide a general interpretation of the results in the context of other evidence. | Y | Y | - | Y |
|  | 23b | Discuss any limitations of the evidence included in the review. | Y | Y | - | Y |
|  | 23c | Discuss any limitations of the review processes used. | Y | Y | - | Y |
|  | 23d | Discuss implications of the results for practice, policy, and future research. | Y | Y | - | Y |
| **Other information** | | | | | | |
| Registration and  protocol | 24a | Provide registration information for the review, including register name and registration number, or state that the review was not registered. | Y | Y | - | Y |
|  | 24b | Indicate where the review protocol can be accessed, or state that a protocol was not prepared. | Y | Y | - | Y |
|  | 24c | Describe and explain any amendments to information provided at registration or in the protocol. | Y | Y | - | Y |
| Support | 25 | Describe sources of financial or non-financial support for the review, and the role of the funders or sponsors in the review. | Y | Y | - | Y |
| Competing interests | 26 | Declare any competing interests of review authors. | Y | Y | - | Y |
| Availability of data,  code, and other  materials | 27 | Report which of the following are publicly available and where they can be found: template data collection forms; data extracted from included studies; data used for all analyses; analytic code; any other materials used in the review. | Y | Y | - | Y |

Table 2：

| Section and topic | Item # | Checklist item | A | B | C | D |
| --- | --- | --- | --- | --- | --- | --- |
| **Title** | | | | | | |
| Title | 1 | Identify the report as a systematic review. | Y | Y | - | Y |
| **Background** | | | | | | |
| Objectives | 2 | Provide an explicit statement of the main objective(s) or question(s) the review addresses. | Y | Y | - | Y |
| **Methods** | | | | | | |
| Eligibility criteria | 3 | Specify the inclusion and exclusion criteria for the review | Y | Y | - | Y |
| Information sources | 4 | Specify the information sources (e.g. databases, registers) used to identify studies and the date when each was last searched. | Y | Y | - | Y |
| Risk of bias | 5 | Specify the methods used to assess risk of bias in the included studies. | Y | Y | - | Y |
| Synthesis of results | 6 | Specify the methods used to present and synthesise results. | Y | Y | - | Y |
| **Results** | | | | | | |
| Included studies | 7 | Give the total number of included studies and participants and summarise relevant characteristics of studies. | Y | Y | - | Y |
| Synthesis of results | 8 | Present results for main outcomes, preferably indicating the number of included studies and participants for each. If meta-analysis was done, report the summary estimate and confidence/credible interval. If comparing groups, indicate the direction of the effect (i.e. which group is favoured). | Y | Y | - | Y |
| **Discussion** | | | | | | |
| Limitations of evidence | 9 | Provide a brief summary of the limitations of the evidence included in the review (e.g. study risk of bias, inconsistency and imprecision). | Y | Y | - | Y |
| Interpretation | 10 | Provide a general interpretation of the results and important implications. | Y | Y | - | Y |
| **Other** | | | | | | |
| Funding | 11 | Specify the primary source of funding for the review. | N | N | - | N |
| Registration | 12 | Provide the register name and registration number. | N | N | - | N |

Study 5：Wang W, Wang X, Wang H, Wang X. Evaluation of Safety, Efficacy, and Compliance of Intralymphatic Immunotherapy for Allergic Rhinoconjunctivitis: A Systematic Review and Meta-Analysis. Int Arch Allergy Immunol. 2023;184(8):754-766. doi: 10.1159/000529025. Epub 2023 Apr 27. PMID: 37105134.

Table 1：

| Section and topic | Item # | Checklist item | A | B | C | D |
| --- | --- | --- | --- | --- | --- | --- |
| **Title** | | | | | | |
| Title | 1 | Identify the report as a systematic review. | Y | Y | - | Y |
| **Abstract** | | | | | | |
| Abstract | 2 | See the PRISMA 2020 for Abstracts checklist (table 2). | PY | PY | - | PY |
| **Introduction** | | | | | | |
| Rationale | 3 | Describe the rationale for the review in the context of existing knowledge | Y | Y | - | Y |
| Objectives | 4 | Provide an explicit statement of the objective(s) or question(s) the review addresses. | Y | Y | - | Y |
| **Methods** | | | | | | |
| Eligibility criteria | 5 | Specify the inclusion and exclusion criteria for the review and how studies were grouped for the syntheses. | Y | Y | - | Y |
| Information sources | 6 | Specify all databases, registers, websites, organisations, reference lists and other sources searched or consulted to identify studies. Specify the date when each source was last searched or consulted. | Y | Y | - | Y |
| Search strategy | 7 | Present the full search strategies for all databases, registers and websites, including any filters and limits used | Y | Y | - | Y |
| Selection process | 8 | Specify the methods used to decide whether a study met the inclusion criteria of the review, including how many reviewers screened each record and each report retrieved, whether they worked independently, and if applicable, details of automation tools  used in the process. | Y | Y | - | Y |
| Data collection  process | 9 | Specify the methods used to collect data from reports, including how many reviewers collected data from each report, whether they worked independently, any processes for obtaining or confirming data from study investigators, and if applicable, details of automation tools used in the process. | Y | Y | - | Y |
| Data items | 10a | List and define all outcomes for which data were sought. Specify whether all results that were compatible with each outcome domain in each study were sought (e.g. for all measures, time points, analyses), and if not, the methods used to decide which  results to collect. | PY | Y | Y | Y |
|  | 10b | List and define all other variables for which data were sought (e.g. participant and intervention characteristics, funding sources). Describe any assumptions made about any missing or unclear information. | PY | Y | Y | Y |
| Study risk of bias  assessment | 11 | Specify the methods used to assess risk of bias in the included studies, including details of the tool(s) used, how many reviewers assessed each study and whether they worked independently, and if applicable, details of automation tools used in the process. | Y | Y | - | Y |
| Effect measures | 12 | Specify for each outcome the effect measure(s) (e.g. risk ratio, mean difference) used in the synthesis or presentation of results. | Y | Y | - | Y |
| Synthesis methods | 13a | Describe the processes used to decide which studies were eligible for each synthesis (e.g. tabulating the study intervention characteristics and comparing against the planned groups for each synthesis (item #5)). | Y | PY | Y | Y |
|  | 13b | Describe any methods required to prepare the data for presentation or synthesis, such as handling of missing summary statistics, or data conversions. | Y | PY | Y | Y |
|  | 13c | Describe any methods used to tabulate or visually display results of individual studies and syntheses. | Y | PY | PY | PY |
|  | 13d | Describe any methods used to synthesise results and provide a rationale for the choice(s). If meta-analysis was performed, describe the model(s), method(s) to identify the presence and extent of statistical heterogeneity, and software package(s) used. | Y | PY | Y | Y |
|  | 13e | Describe any methods used to explore possible causes of heterogeneity among study results (e.g. subgroup analysis, meta regression). | PY | PY | - | PY |
|  | 13f | Describe any sensitivity analyses conducted to assess robustness of the synthesised results. | PY | PY | - | PY |
| Reporting bias  assessment | 14 | Describe any methods used to assess risk of bias due to missing results in a synthesis (arising from reporting biases). | Y | Y | - | Y |
| Certainty assessment | 15 | Describe any methods used to assess certainty (or confidence) in the body of evidence for an outcome. | Y | Y | - | Y |
| **Results** | | | | | | |
| Study selection | 16a | Describe the results of the search and selection process, from the number of records identified in the search to the number of studies included in the review, ideally using a flow diagram (see fig 1). | Y | Y | - | Y |
|  | 16b | Cite studies that might appear to meet the inclusion criteria, but which were excluded, and explain why they were excluded. | Y | Y | - | Y |
| Study characteristics | 17 | Cite each included study and present its characteristics. | Y | Y | - | Y |
| Risk of bias in studies | 18 | Present assessments of risk of bias for each included study. | Y | Y | - | Y |
| Results of individual  studies | 19 | For all outcomes, present, for each study: (a) summary statistics for each group (where appropriate) and (b) an effect estimate and its precision (e.g. confidence/credible interval), ideally using structured tables or plots. | Y | Y | - | Y |
| Results of syntheses | 20a | For each synthesis, briefly summarise the characteristics and risk of bias among contributing studies. | Y | PY | Y | Y |
|  | 20b | Present results of all statistical syntheses conducted. If meta-analysis was done, present for each the summary estimate and its precision (e.g. confidence/credible interval) and measures of statistical heterogeneity. If comparing groups, describe the direction of the effect. | Y | PY | Y | Y |
|  | 20c | Present results of all investigations of possible causes of heterogeneity among study results. | Y | PY | Y | Y |
|  | 20d | Present results of all sensitivity analyses conducted to assess the robustness of the synthesised results. | Y | PY | PY | PY |
| Reporting biases | 21 | Present assessments of risk of bias due to missing results (arising from reporting biases) for each synthesis assessed. | Y | Y | - | Y |
| Certainty of evidence | 22 | Present assessments of certainty (or confidence) in the body of evidence for each outcome assessed. | Y | Y | - | Y |
| **Discussion** | | | | | | |
| Discussion | 23a | Provide a general interpretation of the results in the context of other evidence. | Y | PY | Y | Y |
|  | 23b | Discuss any limitations of the evidence included in the review. | Y | PY | Y | Y |
|  | 23c | Discuss any limitations of the review processes used. | Y | PY | Y | Y |
|  | 23d | Discuss implications of the results for practice, policy, and future research. | Y | PY | Y | Y |
| **Other information** | | | | | | |
| Registration and  protocol | 24a | Provide registration information for the review, including register name and registration number, or state that the review was not registered. | Y | Y | - | Y |
|  | 24b | Indicate where the review protocol can be accessed, or state that a protocol was not prepared. | Y | Y | - | Y |
|  | 24c | Describe and explain any amendments to information provided at registration or in the protocol. | Y | Y | - | Y |
| Support | 25 | Describe sources of financial or non-financial support for the review, and the role of the funders or sponsors in the review. | Y | Y | - | Y |
| Competing interests | 26 | Declare any competing interests of review authors. | Y | Y | - | Y |
| Availability of data,  code, and other  materials | 27 | Report which of the following are publicly available and where they can be found: template data collection forms; data extracted from included studies; data used for all analyses; analytic code; any other materials used in the review. | Y | Y | - | Y |

Table 2：

| Section and topic | Item # | Checklist item | A | B | C | D |
| --- | --- | --- | --- | --- | --- | --- |
| **Title** | | | | | | |
| Title | 1 | Identify the report as a systematic review. | Y | Y | - | Y |
| **Background** | | | | | | |
| Objectives | 2 | Provide an explicit statement of the main objective(s) or question(s) the review addresses. | Y | Y | - | Y |
| **Methods** | | | | | | |
| Eligibility criteria | 3 | Specify the inclusion and exclusion criteria for the review | Y | Y | - | Y |
| Information sources | 4 | Specify the information sources (e.g. databases, registers) used to identify studies and the date when each was last searched. | Y | Y | - | Y |
| Risk of bias | 5 | Specify the methods used to assess risk of bias in the included studies. | Y | Y | - | Y |
| Synthesis of results | 6 | Specify the methods used to present and synthesise results. | Y | Y | - | Y |
| **Results** | | | | | | |
| Included studies | 7 | Give the total number of included studies and participants and summarise relevant characteristics of studies. | Y | Y | - | Y |
| Synthesis of results | 8 | Present results for main outcomes, preferably indicating the number of included studies and participants for each. If meta-analysis was done, report the summary estimate and confidence/credible interval. If comparing groups, indicate the direction of the effect (i.e. which group is favoured). | Y | Y | - | Y |
| **Discussion** | | | | | | |
| Limitations of evidence | 9 | Provide a brief summary of the limitations of the evidence included in the review (e.g. study risk of bias, inconsistency and imprecision). | Y | Y | - | Y |
| Interpretation | 10 | Provide a general interpretation of the results and important implications. | Y | Y | - | Y |
| **Other** | | | | | | |
| Funding | 11 | Specify the primary source of funding for the review. | N | N | - | N |
| Registration | 12 | Provide the register name and registration number. | N | N | - | N |

Study 6：Liu L, Liang Y, Yan L, Li Z. Is intralymphatic immunotherapy effective and safe for allergic rhinitis?: A meta-analysis. Medicine (Baltimore). 2024 Nov 15;103(46):e40589. doi: 10.1097/MD.0000000000040589. PMID: 39560522; PMCID: PMC11576000.

Table 1：

| Section and topic | Item # | Checklist item | A | B | C | D |
| --- | --- | --- | --- | --- | --- | --- |
| **Title** | | | | | | |
| Title | 1 | Identify the report as a systematic review. | Y | Y | - | Y |
| **Abstract** | | | | | | |
| Abstract | 2 | See the PRISMA 2020 for Abstracts checklist (table 2). | PY | PY | - | PY |
| **Introduction** | | | | | | |
| Rationale | 3 | Describe the rationale for the review in the context of existing knowledge | Y | Y | - | Y |
| Objectives | 4 | Provide an explicit statement of the objective(s) or question(s) the review addresses. | Y | Y | - | Y |
| **Methods** | | | | | | |
| Eligibility criteria | 5 | Specify the inclusion and exclusion criteria for the review and how studies were grouped for the syntheses. | Y | Y | - | Y |
| Information sources | 6 | Specify all databases, registers, websites, organisations, reference lists and other sources searched or consulted to identify studies. Specify the date when each source was last searched or consulted. | Y | Y | - | Y |
| Search strategy | 7 | Present the full search strategies for all databases, registers and websites, including any filters and limits used | Y | Y | - | Y |
| Selection process | 8 | Specify the methods used to decide whether a study met the inclusion criteria of the review, including how many reviewers screened each record and each report retrieved, whether they worked independently, and if applicable, details of automation tools  used in the process. | Y | Y | - | Y |
| Data collection  process | 9 | Specify the methods used to collect data from reports, including how many reviewers collected data from each report, whether they worked independently, any processes for obtaining or confirming data from study investigators, and if applicable, details of automation tools used in the process. | Y | Y | - | Y |
| Data items | 10a | List and define all outcomes for which data were sought. Specify whether all results that were compatible with each outcome domain in each study were sought (e.g. for all measures, time points, analyses), and if not, the methods used to decide which  results to collect. | Y | PY | Y | Y |
|  | 10b | List and define all other variables for which data were sought (e.g. participant and intervention characteristics, funding sources). Describe any assumptions made about any missing or unclear information. | Y | Y | - | Y |
| Study risk of bias  assessment | 11 | Specify the methods used to assess risk of bias in the included studies, including details of the tool(s) used, how many reviewers assessed each study and whether they worked independently, and if applicable, details of automation tools used in the process. | Y | Y | - | Y |
| Effect measures | 12 | Specify for each outcome the effect measure(s) (e.g. risk ratio, mean difference) used in the synthesis or presentation of results. | Y | Y | - | Y |
| Synthesis methods | 13a | Describe the processes used to decide which studies were eligible for each synthesis (e.g. tabulating the study intervention characteristics and comparing against the planned groups for each synthesis (item #5)). | Y | Y | - | Y |
|  | 13b | Describe any methods required to prepare the data for presentation or synthesis, such as handling of missing summary statistics, or data conversions. | Y | Y | - | Y |
|  | 13c | Describe any methods used to tabulate or visually display results of individual studies and syntheses. | Y | Y | - | Y |
|  | 13d | Describe any methods used to synthesise results and provide a rationale for the choice(s). If meta-analysis was performed, describe the model(s), method(s) to identify the presence and extent of statistical heterogeneity, and software package(s) used. | Y | Y | - | Y |
|  | 13e | Describe any methods used to explore possible causes of heterogeneity among study results (e.g. subgroup analysis, meta regression). | PY | Y | PY | PY |
|  | 13f | Describe any sensitivity analyses conducted to assess robustness of the synthesised results. | Y | Y | - | Y |
| Reporting bias  assessment | 14 | Describe any methods used to assess risk of bias due to missing results in a synthesis (arising from reporting biases). | Y | Y | - | Y |
| Certainty assessment | 15 | Describe any methods used to assess certainty (or confidence) in the body of evidence for an outcome. | N | PY | PY | PY |
| **Results** | | | | | | |
| Study selection | 16a | Describe the results of the search and selection process, from the number of records identified in the search to the number of studies included in the review, ideally using a flow diagram (see fig 1). | Y | Y | - | Y |
|  | 16b | Cite studies that might appear to meet the inclusion criteria, but which were excluded, and explain why they were excluded. | Y | Y | - | Y |
| Study characteristics | 17 | Cite each included study and present its characteristics. | Y | Y | - | Y |
| Risk of bias in studies | 18 | Present assessments of risk of bias for each included study. | Y | Y | - | Y |
| Results of individual  studies | 19 | For all outcomes, present, for each study: (a) summary statistics for each group (where appropriate) and (b) an effect estimate and its precision (e.g. confidence/credible interval), ideally using structured tables or plots. | Y | Y | - | Y |
| Results of syntheses | 20a | For each synthesis, briefly summarise the characteristics and risk of bias among contributing studies. | Y | Y | - | Y |
|  | 20b | Present results of all statistical syntheses conducted. If meta-analysis was done, present for each the summary estimate and its precision (e.g. confidence/credible interval) and measures of statistical heterogeneity. If comparing groups, describe the direction of the effect. | Y | Y | - | Y |
|  | 20c | Present results of all investigations of possible causes of heterogeneity among study results. | N | PY | PY | PY |
|  | 20d | Present results of all sensitivity analyses conducted to assess the robustness of the synthesised results. | Y | Y | - | Y |
| Reporting biases | 21 | Present assessments of risk of bias due to missing results (arising from reporting biases) for each synthesis assessed. | Y | Y | - | Y |
| Certainty of evidence | 22 | Present assessments of certainty (or confidence) in the body of evidence for each outcome assessed. | Y | PY | PY | PY |
| **Discussion** | | | | | | |
| Discussion | 23a | Provide a general interpretation of the results in the context of other evidence. | Y | Y | - | Y |
|  | 23b | Discuss any limitations of the evidence included in the review. | Y | Y | - | Y |
|  | 23c | Discuss any limitations of the review processes used. | Y | Y | - | Y |
|  | 23d | Discuss implications of the results for practice, policy, and future research. | Y | Y | - | Y |
| **Other information** | | | | | | |
| Registration and  protocol | 24a | Provide registration information for the review, including register name and registration number, or state that the review was not registered. | Y | Y | - | Y |
|  | 24b | Indicate where the review protocol can be accessed, or state that a protocol was not prepared. | Y | Y | - | Y |
|  | 24c | Describe and explain any amendments to information provided at registration or in the protocol. | PY | PY | - | PY |
| Support | 25 | Describe sources of financial or non-financial support for the review, and the role of the funders or sponsors in the review. | N | Y | Y | Y |
| Competing interests | 26 | Declare any competing interests of review authors. | Y | Y | - | Y |
| Availability of data,  code, and other  materials | 27 | Report which of the following are publicly available and where they can be found: template data collection forms; data extracted from included studies; data used for all analyses; analytic code; any other materials used in the review. | Y | N | PY | PY |

Table 2：

| Section and topic | Item # | Checklist item | A | B | C | D |
| --- | --- | --- | --- | --- | --- | --- |
| **Title** | | | | | | |
| Title | 1 | Identify the report as a systematic review. | Y | Y | - | Y |
| **Background** | | | | | | |
| Objectives | 2 | Provide an explicit statement of the main objective(s) or question(s) the review addresses. | Y | Y | - | Y |
| **Methods** | | | | | | |
| Eligibility criteria | 3 | Specify the inclusion and exclusion criteria for the review | Y | Y | - | Y |
| Information sources | 4 | Specify the information sources (e.g. databases, registers) used to identify studies and the date when each was last searched. | Y | Y | - | Y |
| Risk of bias | 5 | Specify the methods used to assess risk of bias in the included studies. | Y | Y | - | Y |
| Synthesis of results | 6 | Specify the methods used to present and synthesise results. | Y | Y | - | Y |
| **Results** | | | | | | |
| Included studies | 7 | Give the total number of included studies and participants and summarise relevant characteristics of studies. | Y | Y | - | Y |
| Synthesis of results | 8 | Present results for main outcomes, preferably indicating the number of included studies and participants for each. If meta-analysis was done, report the summary estimate and confidence/credible interval. If comparing groups, indicate the direction of the effect (i.e. which group is favoured). | Y | Y | - | Y |
| **Discussion** | | | | | | |
| Limitations of evidence | 9 | Provide a brief summary of the limitations of the evidence included in the review (e.g. study risk of bias, inconsistency and imprecision). | Y | Y | - | Y |
| Interpretation | 10 | Provide a general interpretation of the results and important implications. | Y | Y | - | Y |
| **Other** | | | | | | |
| Funding | 11 | Specify the primary source of funding for the review. | N | N | - | N |
| Registration | 12 | Provide the register name and registration number. | N | N | - | N |

Study 7：曾婉婷,李艮平,蒋建国,等.淋巴结内特异性免疫治疗变应性鼻炎疗效和安全性的Meta分析[J].中国耳鼻咽喉颅底外科杂志,2024,30(02):9-18.

Table 1：

| Section and topic | Item # | Checklist item | A | B | C | D |
| --- | --- | --- | --- | --- | --- | --- |
| **Title** | | | | | | |
| Title | 1 | Identify the report as a systematic review. | Y | Y | - | Y |
| **Abstract** | | | | | | |
| Abstract | 2 | See the PRISMA 2020 for Abstracts checklist (table 2). | PY | PY | - | PY |
| **Introduction** | | | | | | |
| Rationale | 3 | Describe the rationale for the review in the context of existing knowledge | Y | Y | - | Y |
| Objectives | 4 | Provide an explicit statement of the objective(s) or question(s) the review addresses. | Y | Y | - | Y |
| **Methods** | | | | | | |
| Eligibility criteria | 5 | Specify the inclusion and exclusion criteria for the review and how studies were grouped for the syntheses. | Y | Y | - | Y |
| Information sources | 6 | Specify all databases, registers, websites, organisations, reference lists and other sources searched or consulted to identify studies. Specify the date when each source was last searched or consulted. | Y | Y | - | Y |
| Search strategy | 7 | Present the full search strategies for all databases, registers and websites, including any filters and limits used | PY | PY | - | PY |
| Selection process | 8 | Specify the methods used to decide whether a study met the inclusion criteria of the review, including how many reviewers screened each record and each report retrieved, whether they worked independently, and if applicable, details of automation tools  used in the process. | PY | Y | Y | Y |
| Data collection  process | 9 | Specify the methods used to collect data from reports, including how many reviewers collected data from each report, whether they worked independently, any processes for obtaining or confirming data from study investigators, and if applicable, details of automation tools used in the process. | PY | Y | Y | Y |
| Data items | 10a | List and define all outcomes for which data were sought. Specify whether all results that were compatible with each outcome domain in each study were sought (e.g. for all measures, time points, analyses), and if not, the methods used to decide which  results to collect. | Y | Y | - | Y |
|  | 10b | List and define all other variables for which data were sought (e.g. participant and intervention characteristics, funding sources). Describe any assumptions made about any missing or unclear information. | Y | Y | - | Y |
| Study risk of bias  assessment | 11 | Specify the methods used to assess risk of bias in the included studies, including details of the tool(s) used, how many reviewers assessed each study and whether they worked independently, and if applicable, details of automation tools used in the process. | PY | Y | Y | Y |
| Effect measures | 12 | Specify for each outcome the effect measure(s) (e.g. risk ratio, mean difference) used in the synthesis or presentation of results. | Y | Y | - | Y |
| Synthesis methods | 13a | Describe the processes used to decide which studies were eligible for each synthesis (e.g. tabulating the study intervention characteristics and comparing against the planned groups for each synthesis (item #5)). | Y | Y | - | Y |
|  | 13b | Describe any methods required to prepare the data for presentation or synthesis, such as handling of missing summary statistics, or data conversions. | PY | Y | Y | Y |
|  | 13c | Describe any methods used to tabulate or visually display results of individual studies and syntheses. | Y | Y | - | Y |
|  | 13d | Describe any methods used to synthesise results and provide a rationale for the choice(s). If meta-analysis was performed, describe the model(s), method(s) to identify the presence and extent of statistical heterogeneity, and software package(s) used. | Y | Y | - | Y |
|  | 13e | Describe any methods used to explore possible causes of heterogeneity among study results (e.g. subgroup analysis, meta regression). | Y | Y | - | Y |
|  | 13f | Describe any sensitivity analyses conducted to assess robustness of the synthesised results. | PY | Y | Y | Y |
| Reporting bias  assessment | 14 | Describe any methods used to assess risk of bias due to missing results in a synthesis (arising from reporting biases). | PY | PY | - | PY |
| Certainty assessment | 15 | Describe any methods used to assess certainty (or confidence) in the body of evidence for an outcome. | PY | Y | PY | PY |
| **Results** | | | | | | |
| Study selection | 16a | Describe the results of the search and selection process, from the number of records identified in the search to the number of studies included in the review, ideally using a flow diagram (see fig 1). | Y | Y | - | Y |
|  | 16b | Cite studies that might appear to meet the inclusion criteria, but which were excluded, and explain why they were excluded. | Y | Y | - | Y |
| Study characteristics | 17 | Cite each included study and present its characteristics. | Y | Y | - | Y |
| Risk of bias in studies | 18 | Present assessments of risk of bias for each included study. | PY | Y | Y | Y |
| Results of individual  studies | 19 | For all outcomes, present, for each study: (a) summary statistics for each group (where appropriate) and (b) an effect estimate and its precision (e.g. confidence/credible interval), ideally using structured tables or plots. | Y | Y | - | Y |
| Results of syntheses | 20a | For each synthesis, briefly summarise the characteristics and risk of bias among contributing studies. | PY | Y | Y | Y |
|  | 20b | Present results of all statistical syntheses conducted. If meta-analysis was done, present for each the summary estimate and its precision (e.g. confidence/credible interval) and measures of statistical heterogeneity. If comparing groups, describe the direction of the effect. | Y | Y | - | Y |
|  | 20c | Present results of all investigations of possible causes of heterogeneity among study results. | Y | Y | - | Y |
|  | 20d | Present results of all sensitivity analyses conducted to assess the robustness of the synthesised results. | PY | Y | Y | Y |
| Reporting biases | 21 | Present assessments of risk of bias due to missing results (arising from reporting biases) for each synthesis assessed. | PY | PY | - | PY |
| Certainty of evidence | 22 | Present assessments of certainty (or confidence) in the body of evidence for each outcome assessed. | PY | Y | Y | Y |
| **Discussion** | | | | | | |
| Discussion | 23a | Provide a general interpretation of the results in the context of other evidence. | Y | Y | - | Y |
|  | 23b | Discuss any limitations of the evidence included in the review. | Y | Y | - | Y |
|  | 23c | Discuss any limitations of the review processes used. | Y | Y | - | Y |
|  | 23d | Discuss implications of the results for practice, policy, and future research. | Y | Y | - | Y |
| **Other information** | | | | | | |
| Registration and  protocol | 24a | Provide registration information for the review, including register name and registration number, or state that the review was not registered. | PY | N | N | N |
|  | 24b | Indicate where the review protocol can be accessed, or state that a protocol was not prepared. | PY | N | N | N |
|  | 24c | Describe and explain any amendments to information provided at registration or in the protocol. | N | N | - | N |
| Support | 25 | Describe sources of financial or non-financial support for the review, and the role of the funders or sponsors in the review. | Y | Y | - | Y |
| Competing interests | 26 | Declare any competing interests of review authors. | Y | Y | - | Y |
| Availability of data,  code, and other  materials | 27 | Report which of the following are publicly available and where they can be found: template data collection forms; data extracted from included studies; data used for all analyses; analytic code; any other materials used in the review. | PY | N | PY | PY |

Table 2：

| Section and topic | Item # | Checklist item | A | B | C | D |
| --- | --- | --- | --- | --- | --- | --- |
| **Title** | | | | | | |
| Title | 1 | Identify the report as a systematic review. | Y | Y | - | Y |
| **Background** | | | | | | |
| Objectives | 2 | Provide an explicit statement of the main objective(s) or question(s) the review addresses. | Y | Y | - | Y |
| **Methods** | | | | | | |
| Eligibility criteria | 3 | Specify the inclusion and exclusion criteria for the review | Y | Y | - | Y |
| Information sources | 4 | Specify the information sources (e.g. databases, registers) used to identify studies and the date when each was last searched. | Y | Y | - | Y |
| Risk of bias | 5 | Specify the methods used to assess risk of bias in the included studies. | Y | Y | - | Y |
| Synthesis of results | 6 | Specify the methods used to present and synthesise results. | Y | Y | - | Y |
| **Results** | | | | | | |
| Included studies | 7 | Give the total number of included studies and participants and summarise relevant characteristics of studies. | Y | Y | - | Y |
| Synthesis of results | 8 | Present results for main outcomes, preferably indicating the number of included studies and participants for each. If meta-analysis was done, report the summary estimate and confidence/credible interval. If comparing groups, indicate the direction of the effect (i.e. which group is favoured). | Y | Y | - | Y |
| **Discussion** | | | | | | |
| Limitations of evidence | 9 | Provide a brief summary of the limitations of the evidence included in the review (e.g. study risk of bias, inconsistency and imprecision). | Y | Y | - | Y |
| Interpretation | 10 | Provide a general interpretation of the results and important implications. | Y | Y | - | Y |
| **Other** | | | | | | |
| Funding | 11 | Specify the primary source of funding for the review. | N | N | - | N |
| Registration | 12 | Provide the register name and registration number. | N | N | - | N |

5.GRADE

A:The conclusions of researcher Ph.D. Zhuang Wang.

B:The conclusions of researcher Ph.D. Xiaofei Xie.

C:In case of a difference of opinions, it shall be adjudicated by Professor Zhikai Qiu.

D:Conclusive conclusion.

①Methodological quality of included studies was low, with biases in randomization, allocation concealment, and blinding. ②The heterogeneity was large and low confidence interval overlap. ③The population was not broadly representative. ④Small sample size, 95% confidence intervals include null values. ⑤Few studies were included, the funnel plot was not symmetrical, Egger’s test found that publication bias or results were positive, and there was no publication bias evaluation.

Study 1：Aini NR, Mohd Noor N, Md Daud MK, Wise SK, Abdullah B. Efficacy and safety of intralymphatic immunotherapy in allergic rhinitis: A systematic review and meta-analysis. Clin Transl Allergy. 2021 Aug 17;11(6):e12055. doi: 10.1002/clt2.12055. PMID: 34429875; PMCID: PMC8369948.

| Endpoint measure | Downgrading factor | A | B | C | D |
| --- | --- | --- | --- | --- | --- |
| Combined symptoms medication score | Risk of bias | -1① | -1① | - | -1① |
|  | Inconsistency | -1② | -1② | - | -1② |
|  | Indirectness | 0 | 0 | - | 0 |
|  | Impression | -1④ | -1④ | - | -1④ |
|  | Publication bias | 0 | 0 | - | 0 |
| Symptoms score | Risk of bias | 0 | 0 | - | 0 |
|  | Inconsistency | -1② | -1② | - | -1② |
|  | Indirectness | 0 | 0 | - | 0 |
|  | Impression | -1④ | -1④ | - | -1④ |
|  | Publication bias | 0 | 0 | - | 0 |
| Medication score | Risk of bias | 0 | 0 | - | 0 |
|  | Inconsistency | -1② | -1② | - | -1② |
|  | Indirectness | 0 | 0 | - | 0 |
|  | Impression | -1④ | -1④ | - | -1④ |
|  | Publication bias | 0 | 0 | - | 0 |
| Rescue medication | Risk of bias | 0 | 0 | - | 0 |
|  | Inconsistency | 0 | 0 | - | 0 |
|  | Indirectness | 0 | 0 | - | 0 |
|  | Impression | -1④ | -1④ | - | -1④ |
|  | Publication bias | -1⑤ | 0 | 0 | 0 |
| Overall improvement score | Risk of bias | 0 | 0 | - | 0 |
|  | Inconsistency | -1② | 0 | -1② | -1② |
|  | Indirectness | 0 | 0 | - | 0 |
|  | Impression | -1④ | -1④ | - | -1④ |
|  | Publication bias | 0 | 0 | - | 0 |
| Local swelling | Risk of bias | 0 | 0 | - | 0 |
|  | Inconsistency | -1② | -1② | - | -1② |
|  | Indirectness | 0 | 0 | - | 0 |
|  | Impression | -1④ | -1④ | - | -1④ |
|  | Publication bias | 0 | 0 | - | 0 |
| Abdominal symptoms | Risk of bias | 0 | 0 | - | 0 |
|  | Inconsistency | 0 | 0 | - | 0 |
|  | Indirectness | 0 | 0 | - | 0 |
|  | Impression | -1④ | -1④ | - | -1④ |
|  | Publication bias | 0 | 0 | - | 0 |
| Nonspecific symptoms | Risk of bias | 0 | 0 | - | 0 |
|  | Inconsistency | 0 | 0 | - | 0 |
|  | Indirectness | 0 | 0 | - | 0 |
|  | Impression | 0 | 0 | - | 0 |
|  | Publication bias | 0 | 0 | - | 0 |
| Eye and nasal symptoms | Risk of bias | 0 | 0 | - | 0 |
|  | Inconsistency | -1② | -1② | - | -1② |
|  | Indirectness | 0 | 0 | - | 0 |
|  | Impression | 0 | 0 | - | 0 |
|  | Publication bias | 0 | 0 | - | 0 |
| Skin prick test | Risk of bias | 0 | -1① | -1① | -1① |
|  | Inconsistency | 0 | 0 | - | 0 |
|  | Indirectness | 0 | 0 | - | 0 |
|  | Impression | -1④ | -1④ | - | -1④ |
|  | Publication bias | 0 | 0 | - | 0 |
| Specific IgE levels | Risk of bias | 0 | 0 | - | 0 |
|  | Inconsistency | 0 | 0 | - | 0 |
|  | Indirectness | 0 | 0 | - | 0 |
|  | Impression | -1④ | -1④ | - | -1④ |
|  | Publication bias | 0 | -1⑤ | 0 | 0 |
| Quality of life | Risk of bias | 0 | 0 | - | 0 |
|  | Inconsistency | -1② | -1② | - | -1② |
|  | Indirectness | 0 | 0 | - | 0 |
|  | Impression | 0 | 0 | - | 0 |
|  | Publication bias | 0 | 0 | - | 0 |

Study 2：Werner MT, Bosso JV. Intralymphatic immunotherapy for allergic rhinitis: A systematic review and meta-analysis. Allergy Asthma Proc. 2021 Jul 1;42(4):283-292. doi: 10.2500/aap.2021.42.210028. PMID: 34187620.

| Endpoint measure | Downgrading factor | A | B | C | D |
| --- | --- | --- | --- | --- | --- |
| Combined symptom and medication scores | Risk of bias | -1① | -1① | - | -1① |
|  | Inconsistency | 0 | 0 | - | 0 |
|  | Indirectness | 0 | 0 | - | 0 |
|  | Impression | 0 | 0 | - | 0 |
|  | Publication bias | -1⑤ | -1⑤ | - | -1⑤ |
| Nasal Provocation Testing | Risk of bias | -1① | -1① | - | -1① |
|  | Inconsistency | 0 | 0 | - | 0 |
|  | Indirectness | 0 | 0 | - | 0 |
|  | Impression | 0 | 0 | - | 0 |
|  | Publication bias | 0 | 0 | - | 0 |
| Skin-Prick Testing | Risk of bias | -1① | -1① | - | -1① |
|  | Inconsistency | 0 | 0 | - | 0 |
|  | Indirectness | 0 | 0 | - | 0 |
|  | Impression | 0 | 0 | - | 0 |
|  | Publication bias | 0 | 0 | - | 0 |
| Overall Adverse Event Rate | Risk of bias | -1① | -1① | - | -1① |
|  | Inconsistency | -1② | -1② | - | -1② |
|  | Indirectness | 0 | 0 | - | 0 |
|  | Impression | -1④ | -1④ | - | -1④ |
|  | Publication bias | -1⑤ | -1⑤ | - | -1⑤ |

Study3：Hoang MP, Seresirikachorn K, Chitsuthipakorn W, Snidvongs K. Intralymphatic immunotherapy for allergic rhinoconjunctivitis: a systematic review and meta-analysis. Rhinology. 2021 Jun 1;59(3):236-244. doi: 10.4193/Rhin20.572. PMID: 33647073.

| Endpoint measure | Downgrading factor | A | B | C | D |
| --- | --- | --- | --- | --- | --- |
| Combined Symptom and Medication Score | Risk of bias | -1① | -1① | - | -1① |
|  | Inconsistency | 0 | 0 | - | 0 |
|  | Indirectness | 0 | 0 | - | 0 |
|  | Impression | -1④ | 0 | -1④ | -1④ |
|  | Publication bias | 0 | 0 | - | 0 |
| Visual Analog Scale | Risk of bias | -1① | -1① | - | -1① |
|  | Inconsistency | 0 | 0 | - | 0 |
|  | Indirectness | 0 | 0 | - | 0 |
|  | Impression | 0 | 0 | - | 0 |
|  | Publication bias | 0 | 0 | - | 0 |
| Disease-specific Quality of Life | Risk of bias | -1① | -1① | - | -1① |
|  | Inconsistency | -1② | -1② | - | -1② |
|  | Indirectness | 0 | 0 | - | 0 |
|  | Impression | -1④ | -1④ | - | -1④ |
|  | Publication bias | 0 | -1⑤ | 0 | 0 |
| Specific IgG4 Level | Risk of bias | -1① | -1① | - | -1① |
|  | Inconsistency | -1② | 0 | -1② | -1② |
|  | Indirectness | 0 | 0 | - | 0 |
|  | Impression | -1④ | -1④ | - | -1④ |
|  | Publication bias | 0 | 0 | - | 0 |
| Specific IgE Level | Risk of bias | -1① | -1① | - | -1① |
|  | Inconsistency | -1② | 0 | 0 | 0 |
|  | Indirectness | 0 | 0 | - | 0 |
|  | Impression | 0 | 0 | - | 0 |
|  | Publication bias | 0 | 0 | - | 0 |
| Local reactions of Adverse Events | Risk of bias | -1① | -1① | - | -1① |
|  | Inconsistency | 0 | 0 | - | 0 |
|  | Indirectness | 0 | 0 | - | 0 |
|  | Impression | -1④ | -1④ | - | -1④ |
|  | Publication bias | 0 | 0 | - | 0 |
| Systemic reactions of Adverse Events | Risk of bias | -1① | -1① | - | -1① |
|  | Inconsistency | 0 | 0 | - | 0 |
|  | Indirectness | 0 | 0 | - | 0 |
|  | Impression | -1④ | -1④ | - | -1④ |
|  | Publication bias | 0 | 0 | - | 0 |
| Symptom Score | Risk of bias | -1① | -1① | - | -1① |
|  | Inconsistency | 0 | 0 | - | 0 |
|  | Indirectness | 0 | 0 | - | 0 |
|  | Impression | -1④ | -1④ | - | -1④ |
|  | Publication bias | 0 | 0 | - | 0 |
| Medication Score | Risk of bias | -1① | -1① | - | -1① |
|  | Inconsistency | 0 | 0 | - | 0 |
|  | Indirectness | -1③ | 0 | 0 | 0 |
|  | Impression | -1④ | -1④ | - | -1④ |
|  | Publication bias | 0 | 0 | - | 0 |

Study 4：Jiang S, Xie S, Tang Q, Zhang H, Xie Z, Zhang J, Jiang W. Evaluation of Intralymphatic Immunotherapy in Allergic Rhinitis Patients: A Systematic Review and Meta-analysis. Mediators Inflamm. 2023 May 8;2023.

| Endpoint measure | Downgrading factor | A | B | C | D |
| --- | --- | --- | --- | --- | --- |
| Combined Symptom and Medication Scores | Risk of bias | -1① | -1① | - | -1① |
|  | Inconsistency | -1② | -1② | - | -1② |
|  | Indirectness | 0 | 0 | - | 0 |
|  | Impression | -1④ | -1④ | - | -1④ |
|  | Publication bias | 0 | 0 | - | 0 |
| Allergic Rhinoconjunctivitis Quality of Life | Risk of bias | 0 | 0 | - | 0 |
|  | Inconsistency | 0 | 0 | - | 0 |
|  | Indirectness | 0 | -1③ | 0 | 0 |
|  | Impression | 0 | 0 | - | 0 |
|  | Publication bias | 0 | 0 | - | 0 |
| Visual Analog Scale | Risk of bias | -1① | -1① | - | -1① |
|  | Inconsistency | -1② | -1② | - | -1② |
|  | Indirectness | 0 | 0 | - | 0 |
|  | Impression | -1④ | -1④ | - | -1④ |
|  | Publication bias | 0 | 0 | - | 0 |
| The impact of booster injection on CSMs | Risk of bias | -1① | -1① | - | -1① |
|  | Inconsistency | -1② | -1② | - | -1② |
|  | Indirectness | 0 | 0 | - | 0 |
|  | Impression | 0 | 0 | - | 0 |
|  | Publication bias | 0 | 0 | - | 0 |
| The impact of no booster injection on CSMs | Risk of bias | -1① | -1① | - | -1① |
|  | Inconsistency | -1② | -1② | - | -1② |
|  | Indirectness | 0 | 0 | - | 0 |
|  | Impression | -1④ | -1④ | - | -1④ |
|  | Publication bias | 0 | -1⑤ | -1⑤ | -1⑤ |
| The impact of 4-week injection interval on CSMs | Risk of bias | -1① | -1① | - | -1① |
|  | Inconsistency | -1② | -1② | - | -1② |
|  | Indirectness | 0 | 0 | - | 0 |
|  | Impression | -1④ | -1④ | - | -1④ |
|  | Publication bias | 0 | 0 | - | 0 |
| The impact of 2-week injection interval on CSMs | Risk of bias | -1① | -1① | - | -1① |
|  | Inconsistency | -1② | -1② | - | -1② |
|  | Indirectness | 0 | 0 | - | 0 |
|  | Impression | -1④ | -1④ | - | -1④ |
|  | Publication bias | 0 | 0 | - | 0 |
| The impact of 4-week injection interval on VAS | Risk of bias | -1① | -1① | - | -1① |
|  | Inconsistency | 0 | 0 | - | 0 |
|  | Indirectness | 0 | 0 | - | 0 |
|  | Impression | 0 | 0 | - | 0 |
|  | Publication bias | 0 | 0 | - | 0 |
| The impact of 2-week injection interval on VAS | Risk of bias | -1① | -1① | - | -1① |
|  | Inconsistency | -1② | -1② | - | -1② |
|  | Indirectness | 0 | -1③ | 0 | 0 |
|  | Impression | -1④ | -1④ | - | -1④ |
|  | Publication bias | 0 | -1⑤ | -1⑤ | -1⑤ |
| Skin-Prick Test | Risk of bias | 0 | 0 | - | 0 |
|  | Inconsistency | 0 | 0 | - | 0 |
|  | Indirectness | 0 | 0 | - | 0 |
|  | Impression | 0 | 0 | - | 0 |
|  | Publication bias | 0 | 0 | - | 0 |
| Adverse Events | Risk of bias | -1① | -1① | - | -1① |
|  | Inconsistency | -1② | -1② | - | -1② |
|  | Indirectness | 0 | 0 | - | 0 |
|  | Impression | -1④ | -1④ | - | -1④ |
|  | Publication bias | 0 | 0 | - | 0 |

Study 5：Wang W, Wang X, Wang H, Wang X. Evaluation of Safety, Efficacy, and Compliance of Intralymphatic Immunotherapy for Allergic Rhinoconjunctivitis: A Systematic Review and Meta-Analysis. Int Arch Allergy Immunol. 2023;184(8):754-766. doi: 10.1159/000529025. Epub 2023 Apr 27. PMID: 37105134.

| Endpoint measure | Downgrading factor | A | B | C | D |
| --- | --- | --- | --- | --- | --- |
| Local Adverse Events | Risk of bias | 0 | 0 | - | 0 |
|  | Inconsistency | -1② | -1② | - | -1② |
|  | Indirectness | 0 | 0 | - | 0 |
|  | Impression | -1④ | -1④ | - | -1④ |
|  | Publication bias | 0 | 0 | - | 0 |
| Systemic Adverse Events | Risk of bias | 0 | 0 | - | 0 |
|  | Inconsistency | -1② | -1② | - | -1② |
|  | Indirectness | 0 | 0 | - | 0 |
|  | Impression | -1④ | -1④ | - | -1④ |
|  | Publication bias | 0 | 0 | - | 0 |
| Proportion of subjects experiencing AEs | Risk of bias | 0 | 0 | - | 0 |
|  | Inconsistency | 0 | 0 | - | 0 |
|  | Indirectness | 0 | 0 | - | 0 |
|  | Impression |  |  |  |  |
|  | Publication bias |  |  |  |  |
| Compliance | Risk of bias | 0 | -1① | -1① | -1① |
|  | Inconsistency | -1② | -1② | - | -1② |
|  | Indirectness | 0 | 0 | - | 0 |
|  | Impression | -1④ | 0 | 0 | 0 |
|  | Publication bias | 0 | 0 | - | 0 |
| Symptom and Medication Score | Risk of bias | 0 | 0 | - | 0 |
|  | Inconsistency | -1② | -1② | - | -1② |
|  | Indirectness | -1③ | -1③ | - | -1③ |
|  | Impression | 0 | 0 | - | 0 |
|  | Publication bias | 0 | 0 | - | 0 |
| Seasonal ARC | Risk of bias | 0 | 0 | - | 0 |
|  | Inconsistency | 0 | 0 | - | 0 |
|  | Indirectness | 0 | 0 | - | 0 |
|  | Impression | -1④ | 0 | -1④ | -1④ |
|  | Publication bias | 0 | 0 | - | 0 |
| 4-Week Interval | Risk of bias | 0 | 0 | - | 0 |
|  | Inconsistency | 0 | 0 | - | 0 |
|  | Indirectness | -1④ | 0 | 0 | 0 |
|  | Impression | 0 | 0 | - | 0 |
|  | Publication bias | 0 | 0 | - | 0 |
| Same Doses | Risk of bias | 0 | 0 | - | 0 |
|  | Inconsistency | 0 | 0 | - | 0 |
|  | Indirectness | 0 | 0 | - | 0 |
|  | Impression | 0 | 0 | - | 0 |
|  | Publication bias | 0 | 0 | - | 0 |
| Escalating Doses | Risk of bias | 0 | 0 | - | 0 |
|  | Inconsistency | -1② | -1② | - | -1② |
|  | Indirectness | 0 | 0 | - | 0 |
|  | Impression | -1④ | 0 | -1④ | -1④ |
|  | Publication bias | 0 | 0 | - | 0 |

Study 6：Liu L, Liang Y, Yan L, Li Z. Is intralymphatic immunotherapy effective and safe for allergic rhinitis?: A meta-analysis. Medicine (Baltimore). 2024 Nov 15;103(46):e40589. doi: 10.1097/MD.0000000000040589. PMID: 39560522; PMCID: PMC11576000.

| Endpoint measure | Downgrading factor | A | B | C | D |
| --- | --- | --- | --- | --- | --- |
| Symptom Score | Risk of bias | -1① | -1① | - | -1① |
|  | Inconsistency | 0 | 0 | - | 0 |
|  | Indirectness | -1③ | -1③ | - | -1③ |
|  | Impression | -1④ | -1④ | - | -1④ |
|  | Publication bias | 0 | 0 | - | 0 |
| Medication Score | Risk of bias | -1① | -1① | - | -1① |
|  | Inconsistency | -1② | -1② | - | -1② |
|  | Indirectness | -1③ | -1③ | - | -1③ |
|  | Impression | -1④ | -1④ | - | -1④ |
|  | Publication bias | 0 | 0 | - | 0 |
| Comprehensive Symptom and Medication Score | Risk of bias | -1① | -1① | - | -1① |
|  | Inconsistency | -1② | -1② | - | -1② |
|  | Indirectness | -1③ | -1③ | - | -1③ |
|  | Impression | -1④ | -1④ | - | -1④ |
|  | Publication bias | 0 | 0 | - | 0 |
| Nasal Symptoms | Risk of bias | 0 | 0 | - | 0 |
|  | Inconsistency | 0 | -1② | 0 | 0 |
|  | Indirectness | 0 | -1③ | -1③ | -1③ |
|  | Impression | 0 | 0 | - | 0 |
|  | Publication bias | 0 | 0 | - | 0 |
| Lymphadenopathy | Risk of bias | 0 | 0 | - | 0 |
|  | Inconsistency | 0 | 0 | - | 0 |
|  | Indirectness | -1③ | -1③ | - | -1③ |
|  | Impression | -1④ | -1④ | - | -1④ |
|  | Publication bias | 0 | 0 | - | 0 |
| Quality of Life | Risk of bias | -1① | -1① | - | -1① |
|  | Inconsistency | 0 | 0 | - | 0 |
|  | Indirectness | -1③ | 0 | -1③ | -1③ |
|  | Impression | 0 | -1④ | 0 | 0 |
|  | Publication bias | 0 | 0 | - | 0 |
| Immunoglobulin E | Risk of bias | -1① | -1① | - | -1① |
|  | Inconsistency | 0 | 0 | - | 0 |
|  | Indirectness | -1③ | -1③ | - | -1③ |
|  | Impression | -1④ | -1④ | - | -1④ |
|  | Publication bias | 0 | 0 | - | 0 |

Study 7：曾婉婷,李艮平,蒋建国,等.淋巴结内特异性免疫治疗变应性鼻炎疗效和安全性的Meta分析[J].中国耳鼻咽喉颅底外科杂志,2024,30(02):9-18.

| Endpoint measure | Downgrading factor | A | B | C | D |
| --- | --- | --- | --- | --- | --- |
| Symptom Score | Risk of bias | -1① | -1① | - | -1① |
|  | Inconsistency | 0 | 0 | - | 0 |
|  | Indirectness | -1③ | -1③ | - | -1③ |
|  | Impression | -1④ | -1④ | - | -1④ |
|  | Publication bias | 0 | 0 | - | 0 |
| Medication Score | Risk of bias | -1① | -1① | - | -1① |
|  | Inconsistency | 0 | 0 | - | 0 |
|  | Indirectness | -1③ | -1③ | - | -1③ |
|  | Impression | -1④ | -1④ | - | -1④ |
|  | Publication bias | 0 | 0 | - | 0 |
| Combined Symptom and Medication Score | Risk of bias | -1① | -1① | - | -1① |
|  | Inconsistency | -1② | 0 | -1② | -1② |
|  | Indirectness | -1③ | -1③ | - | -1③ |
|  | Impression | -1④ | -1④ | - | -1④ |
|  | Publication bias | 0 | 0 | - | 0 |
| Visual Analog Scale | Risk of bias | -1① | -1① | - | -1① |
|  | Inconsistency | 0 | 0 | - | 0 |
|  | Indirectness | -1③ | -1③ | - | -1③ |
|  | Impression | -1④ | -1④ | - | -1④ |
|  | Publication bias | 0 | 0 | - | 0 |
| Quality of Life | Risk of bias | -1① | -1① | - | -1① |
|  | Inconsistency | -1② | -1② | - | -1② |
|  | Indirectness | -1③ | -1③ | - | -1③ |
|  | Impression | -1④ | -1④ | - | -1④ |
|  | Publication bias | 0 | 0 | - | 0 |
| The short-term efficacy of Serum Specific IgE | Risk of bias | -1① | -1① | - | -1① |
|  | Inconsistency | 0 | 0 | - | 0 |
|  | Indirectness | -1③ | -1③ | - | -1③ |
|  | Impression | -1④ | -1④ | - | -1④ |
|  | Publication bias | 0 | 0 | - | 0 |
| The medium-term efficacy of Serum Specific IgE | Risk of bias | 0 | -1① | -1① | -1① |
|  | Inconsistency | -1② | 0 | 0 | 0 |
|  | Indirectness | -1③ | -1③ | - | -1③ |
|  | Impression | -1④ | -1④ | - | -1④ |
|  | Publication bias | 0 | 0 | - | 0 |
| The long-term efficacy of Serum Specific IgE | Risk of bias | -1① | -1① | - | -1① |
|  | Inconsistency | 0 | 0 | - | 0 |
|  | Indirectness | -1③ | -1③ | - | -1③ |
|  | Impression | -1④ | -1④ | - | -1④ |
|  | Publication bias | 0 | 0 | - | 0 |
| The short-term efficacy of Serum Specific IgG4 | Risk of bias | -1① | -1① | - | -1① |
|  | Inconsistency | 0 | 0 | - | 0 |
|  | Indirectness | -1③ | -1③ | - | -1③ |
|  | Impression | -1④ | -1④ | - | -1④ |
|  | Publication bias | 0 | 0 | - | 0 |
| The medium-term efficacy of Serum Specific IgG4 | Risk of bias | -1① | -1① | - | -1① |
|  | Inconsistency | 0 | 0 | - | 0 |
|  | Indirectness | -1③ | -1③ | - | -1③ |
|  | Impression | -1④ | -1④ | - | -1④ |
|  | Publication bias | 0 | 0 | - | 0 |
| The long-term efficacy of Serum Specific IgG4 | Risk of bias | -1① | -1① | - | -1① |
|  | Inconsistency | 0 | 0 | - | 0 |
|  | Indirectness | -1③ | -1③ | - | -1③ |
|  | Impression | -1④ | -1④ | - | -1④ |
|  | Publication bias | 0 | 0 | - | 0 |
| Skin Prick Test | Risk of bias | 0 | -1① | -1① | -1① |
|  | Inconsistency | 0 | -1② | 0 | 0 |
|  | Indirectness | -1③ | -1③ | - | -1③ |
|  | Impression | -1④ | -1④ | - | -1④ |
|  | Publication bias | 0 | 0 | - | 0 |

6.Excluded literature and the reasons for exclusion

**Duplicate literature**

1.Does evidence support the use of cat allergen immunotherapy?

2.Efficacy and safety of intralymphatic immunotherapy in allergic rhinitis: A systematic review and meta-analysis

3.Efficacy and safety of intralymphatic immunotherapy in allergic rhinitis: A systematic review and meta-analysis

4.Evaluation of Intralymphatic Immunotherapy in Allergic Rhinitis Patients: A Systematic Review and Meta-analysis

5.Evaluation of Intralymphatic Immunotherapy in Allergic Rhinitis Patients: A Systematic Review and Meta-analysis

6.Intralymphatic immunotherapy for allergic rhinitis: A systematic review and meta-analysis

7.Intralymphatic immunotherapy for allergic rhinitis: A systematic review and meta-analysis

8.Is intralymphatic immunotherapy effective and safe for allergic rhinitis? A meta-analysis

9.Is intralymphatic immunotherapy effective and safe for allergic rhinitis? A meta-analysis

10.A meta-analysis investigating the efficacy and safety of allergen-specific immunotherapy in the management of respiratory allergies

11.Evaluation of Safety, Efficacy, and Compliance of Intralymphatic Immunotherapy for Allergic Rhinoconjunctivitis: A Systematic Review and Meta-Analysis

12.Evaluation of Safety, Efficacy, and Compliance of Intralymphatic Immunotherapy for Allergic Rhinoconjunctivitis: A Systematic Review and Meta-Analysis

13.Intralymphatic immunotherapy for allergic rhinoconjunctivitis: a systematic review and meta-analysis

14.Intralymphatic immunotherapy for allergic rhinoconjunctivitis: a systematic review and meta-analysis

15.淋巴结内特异性免疫治疗变应性鼻炎疗效和安全性的Meta分析

16.淋巴结内特异性免疫治疗变应性鼻炎疗效和安全性的Meta分析

17.淋巴结内特异性免疫治疗变应性鼻炎疗效和安全性的Meta分析

**Non-allergic rhinitis or cases where allergic rhinitis or allergic rhinoconjunctivitis is not the main disease for treatment**

1.2009年至2018年我国2364例自身免疫性胰腺炎患者临床特征分析

2.变应原特异性免疫治疗在变应性鼻炎伴哮喘治疗中的临床疗效

3.蜂房儿科临床应用举隅

4.毛细支气管炎临床分析及临床路径实施效果评价

5.难治性咽异感症相关病因分析

7.薛伯寿教授应用升降散方证规律及临床传承研究

**Non-Intralymphatic immunotherapy in the treatment group or non-placebo/Subcutaneous Immunotherapy therapy in the control group**

1.Allergen immunotherapy for allergic rhinoconjunctivitis: A systematic review and meta-analysis

2.奥洛他定—莫米松联合鼻喷雾剂及不同免疫疗法治疗变应性鼻炎疗效和安全性的meta分析

3.变应性鼻炎吸入性和食入性变应原特异性免疫球蛋白E检测分析

4.沧州市区气传花粉监测及其与变应性鼻炎吸入性变应原谱的相关性研究

5.过敏性鼻炎患儿变应原特异性免疫治疗前后炎症因子表达谱变化及其意义

6.基于系统药理学方法探讨散风通窍滴丸治疗过敏性鼻炎的作用靶点及机制

7.Der f2重组蛋白用于变应性鼻炎患者血清ELISA检测

**Review**

1.New approaches to allergen immunotherapy

2.儿童变应性鼻炎变应原特异性免疫治疗的疗效评价

**Unable to acquire complete data**

1.Efficacy of intralymphatic immunotherapy in allergic rhinitis: A systematic review and meta-analysis

**Deviation from the research theme**

1.Allergen immunotherapy for allergic rhinoconjunctivitis: A systematic review and meta-analysis

2.Allergen immunotherapy in allergic rhinitis: current use and future trends

3.A comparison of immunotherapy delivery methods for allergen immunotherapy

4.Does evidence support the use of cat allergen immunotherapy?

5.Investigational new drugs for allergic rhinitis

6.Mechanisms of allergen-specific immunotherapy and allergen tolerance

7.A meta-analysis investigating the efficacy and safety of allergen-specific immunotherapy in the management of respiratory allergies

8.Novel strategies for the treatment of grass pollen-induced allergic rhinitis

9.Strengthening the case for intralymphatic immunotherapy

10.20th Malaysian Society of Allergy and Immunology

11.颈部淋巴结内特异性免疫治疗变应性鼻炎疗效及安全性的研究
